# Combination of PKCδ Inhibition with Conventional TKI Treatment to Target CML Models

**DOI:** 10.3390/cancers13071693

**Published:** 2021-04-02

**Authors:** Fabien Muselli, Lucas Mourgues, Rita Morcos, Nathalie Rochet, Marielle Nebout, Agnès Guerci-Bresler, Douglas V Faller, Robert M William, Rana Mhaidly, Els Verhoeyen, Laurence Legros, Jean-François Peyron, Didier Mary

**Affiliations:** 1 Université Côte d’Azur, Institut National de la Santé et de la Recherche Médicale (Inserm) U1065, Centre Méditerranéen de Médecine Moléculaire (C3M), 06204 Nice, France; fabien.muselli@univ-cotedazur.fr (F.M.); lmourgues@unice.fr (L.M.); rita-morcos@hotmail.com (R.M.); marielle.nebout@univ-cotedazur.fr (M.N.); rana.mhaidly@univ-cotedazur.fr (R.M.); els.verhoeyen@univ-cotedazur.fr (E.V.); jean-francois.peyron@univ-cotedazur.fr (J.-F.P.); 2 Institut de Biologie Valrose, Université Côte d’Azur, CNRS UMR 7277, Inserm U1091, CEDEX 02, 06107 Nice, France; nathalie.rochet@univ-cotedazur.fr; 3 Hematology Department, University Hospital, 54035 Nancy, France; a.guerci@chru-nancy.fr; 4 Oncology Clinical Research, Millennium Pharmaceuticals Inc., 40 Landsdowne Street, Cambridge, MA 02139, USA; dfaller@bu.edu; 5 Equipe labellisée Ligue Contre le Cancer, 06204 Nice, France; 6 CIRI–International Center for Infectiology Research, Université Claude Bernard Lyon 1, CNRS, UMR5308, Ecole Normale Supérieure de Lyon, 69007 Lyon, France; 7 Department of Hematology, AP-HP Paul Brousse, 94800 Villejuif, France; laurence.legros@aphp.fr

**Keywords:** chronic myeloid leukemia, leukemic stem cells, tyrosine kinase inhibitors, apoptosis, BMI1, PKCδ

## Abstract

**Simple Summary:**

The tyrosine kinase inhibitor (TKI) imatinib was the first targeted therapy to show clinical efficacy against chronic myeloid leukemia (CML) through inhibition of the breakpoint cluster region–Abelson murine leukemia viral oncogene homolog (BCR-ABL), which is responsible for the disease. Two other generations of TKIs have succeeded imatinib, offering additional therapeutic solutions for a growing number of patients with imatinib-resistant CML. However, these clinical approaches although very effective, generate many unwanted side effects because of their daily administration. Attempts to stop TKI when the disease is no longer detectable at the molecular level, unfortunately result in relapses in more than half of cases. This highlights the presence of undetectable leukemia cells, recognized as leukemic stem cells (LSCs) that are TKI insensitive. It therefore appears necessary to identify new biochemical pathways in LSCs, the targeting of which would make re-sensitization to TKIs possible. The results presented here demonstrate that targeting the protein kinase Cδ (PKCδ) pathway represents a valid alternative for LSC elimination.

**Abstract:**

Numerous combinations of signaling pathway blockades in association with tyrosine kinase inhibitor (TKI) treatment have been proposed for eradicating leukemic stem cells (LSCs) in chronic myeloid leukemia (CML), but none are currently clinically available. Because targeting protein kinase Cδ (PKCδ) was demonstrated to eliminate cancer stem cells (CSCs) in solid tumors, we evaluated the efficacy of PKCδ inhibition in combination with TKIs for CML cells. We observed that inhibition of PKCδ by a pharmacological inhibitor, by gene silencing, or by using K562 CML cells expressing dominant-negative (DN) or constitutively active (CA) PKCδ isoforms clearly points to PKCδ as a regulator of the expression of the stemness regulator BMI1. As a consequence, inhibition of PKCδ impaired clonogenicity and cell proliferation for leukemic cells. PKCδ targeting in K562 and LAMA-84 CML cell lines clearly enhanced the apoptotic response triggered by any TKI. A strong synergism was observed for apoptosis induction through an increase in caspase-9 and caspase-3 activation and significantly decreased expression of the Bcl-xL Bcl-2 family member. Inhibition of PKCδ did not modify BCR-ABL phosphorylation but acted downstream of the oncogene by downregulating BMI1 expression, decreasing clonogenicity. PKCδ inhibition interfered with the clonogenicity of primary CML CD34^+^ and BCR-ABL-transduced healthy CD34^+^ cells as efficiently as any TKI while it did not affect differentiation of healthy CD34^+^ cells. LTC-IC experiments pinpointed that PKCδ inhibition strongly decreased the progenitors/LSCs frequency. All together, these results demonstrate that targeting of PKCδ in combination with a conventional TKI could be a new therapeutic opportunity to affect for CML cells.

## 1. Introduction

The management of patients with chronic myeloid leukemia (CML) was transformed with the implementation of tyrosine kinase inhibitors (TKIs) to target the breakpoint cluster region-Abelson murine leukemia viral oncogene homolog (BCR-ABL), which is responsible for the disease. Since the first use of the TKI imatinib [1,2], two other generations of TKIs have followed, widening the treatment possibilities [3]. The different TKIs can be distinguished as mono or dual TKIs; the latter targets both BCR-ABL and Src kinases [4,5,6]. Despite significant success, nearly half of patients with undetectable BCR-ABL levels for whom treatment is discontinued to decrease side effects experience a relapse of the leukemia [7]. A cure for CML is believed to be achievable only after eradicating the persistent therapy-resistant cells in the body, namely, leukemic stem cells (LSCs) or leukemia initiating cells (LICs) [8,9]. Numerous studies have emerged during the last decade demonstrating the need to specifically target LSCs/LICs in order to prevent CML relapse (see [10] for a review).

Therefore, the only way to cure CML is, probably, to eliminate not only differentiated mature leukemia cells, leukemia progenitors, but also, most importantly, LSCs. Indeed, it is well known that although the bulk of chronic myeloid leukemia cells are very sensitive to BCR-ABL inhibitors, CML LSCs are not dependent on BCR-ABL kinase activity to survive [11,12]. The existence of a pool of drug-resistant quiescent LSCs accounts for the failure of full therapy in CML, and the specific targeting of these cells remains to be realized. As LSCs do not express specific antigens at their surfaces, most targeting strategies have focused on the biochemical pathways crucial for their persistence [10]. Some of these pathways depend on the bone marrow microenvironment to control LSC quiescence and survival. Pharmacological approaches have shown efficacy on the miR-300/protein phosphatase 2A (PP2A) axis [13]. The quiescence and persistence of LSCs were also described to be consequences of miR-126 upregulation after TKI treatment, and treatment with an miR-126 inhibitor was proposed as a new option for the elimination of LSCs [14]. The idea of bringing LSCs out of quiescence to resensitize them to TKIs has already been demonstrated using the Peroxisome Proliferator-activated Receptor gamma (PPARγ) agonist pioglitazone, which decreases the expression of signal transducer and activator of transcription 5 (STAT5) [15].

The problem of persistent and insensitive primitive cancer stem cells (CSCs) associated with chemotherapy resistance and relapse is not restricted to hematological disorders. Many studies during the last decade have highlighted the potential of targeting the protein kinase C (PKC) family to affect persistent and drug-insensitive cancer stem cells (CSCs), which are associated with chemotherapy resistance and relapse in various solid cancers, apart from hematological disorders [16,17].

The PKC family includes 10 isoforms that show differential tissue expression and can even display opposite biological effects in different cell types [18]. Interestingly, numerous studies have shown a potential to target individual isoforms in a cancer-specific manner, such as PKCα in breast cancer [19] or PKCε in glioma [20]. The PKCδ isoform is ubiquitously expressed and can play an antiapoptotic role or, conversely, an oncogenic role, depending on the cellular system and apoptotic stimuli [21]. Interestingly, the survival of CSCs in breast, prostatic, melanoma, and pancreatic cancer was reported to decrease after the targeting of PKCδ [22].

Because it was recently reported that targeting PKCδ can resensitize CSCs to TKIs in epithelial growth factor receptor (EGFR)-mutant lung cancer [23], we explored this possibility in the context of CML. We chose the inhibitor BJE6 over rottlerin and sotrastaurin because of its higher PKCδ selectivity and harmlessness towards normal cells [22]. Rottlerin has recently also been described to act via PKCδ-independent pathways [24,25] and synergize with imatinib to induce CML cell death independently of PKCδ. Sotrastaurin targets PKCδ isoforms such as PKCα and PKCβ [19]. Here, we demonstrate that PKCδ inhibition first decreased the expression of BMI1, which is crucial for the self-renewal of LSCs, and subsequently sensitized both proliferative and primary CML CD34^+^ cells to all the clinically available TKIs.

## 2. Results

### 2.1. BCR-ABL Controls BMI1 Expression by Increasing PKCδ Levels in CML Cells

**Figure 1:**
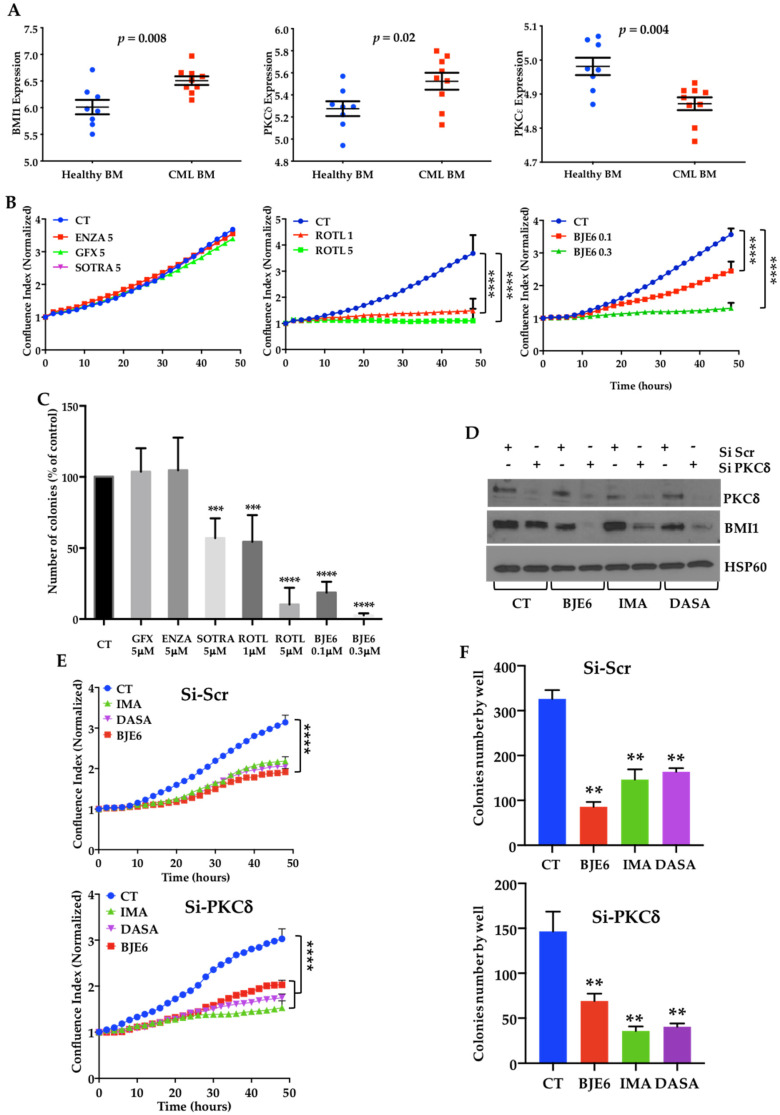
Targeting protein kinase Cδ (PKCδ) inhibited proliferation and clonogenicity of K562 cells. (**A**) BMI1, PKCδ, and PKCε gene expression in bone marrow CD34^+^ cells from healthy donors and chronic myeloid leukemia (CML) patients (GSE5550). (**B**) Time-lapse proliferation analysis of K562 cells treated for 48 h with Dimethyl Sulfoxide (DMSO) (CT) and 5 μM enzastaurin (ENZA), sotrastaurin (SOTRA), and GF109203X (GFX) (left panel); 1 and 5 μM rottlerin (ROTL) (middle panel); and 0.1 and 0.3 μM BJE6-106 (BJE6) (right panel). (**C**) Clonogenic analysis of K562 cells treated for seven days in the same conditions as (**B**). Results expressed as % of control (388 ± 17%). ** *p* < 0.01, *** *p* < 0.001, **** *p* < 0.0001, two-way ANOVA. (**D**) K562 cells transfected with 20 nM PKCδ-siRNA or scr-siRNA and treated for 24 h with DMSO (CT), 0.5 μM BJE6, 1 μM imatinib (IMA), or 2 nM dasatinib (DASA). Cell lysates were analyzed by immunoblotting for indicated proteins. (**E**) Time-lapse proliferation analysis of K562 cells transfected with 20 nM PKCδ-siRNA or scr-siRNA and treated for 48 h with DMSO (CT), 0.1 μM BJE6, 1 μM IMA, or 2 nM DASA. (**F**) Clonogenic analysis of K562 cells treated for 7 days in the same conditions as (**D**). Time-lapse analysis of cells performed with IncuCyte system. Graphs show quantification of cell numbers from phase-contrast confluence. Data represent mean ± SD (*n* = 3). **** *p* < 0.0001, two-way ANOVA, Tukey’s multiple comparison. A.U., arbitrary units.

We previously reported that CML progression is associated with an increase in BMI1 gene expression [26]. The gene expression profiling of bone marrow CD34^+^ cells confirmed higher BMI1 expression in CML patients than in healthy donors (*p* = 0.008), and this was associated with an increase in PKCδ mRNA expression (*p* = 0.02), while PKCε mRNA decreased (*p* = 0.004) (Figure 1A). We then compared different PKC inhibitors for their effects on the proliferation (Figure 1B) and clonogenicity (Figure 1C) of the K562 CML cell line. Neither GF109203X (*GFX*), a potent inhibitor of PKCα, PKCβ, and PKCγ, nor enzastaurin (ENZA), which inhibits PKCα, PKCβ, PKCγ, and PKCε, decreased the proliferation or clonogenicity of the K562 cells at 5 µM. At the same dose, sotrastaurin (SOTRA), which inhibits PKCδ in addition to PKCθ, PKCβ, PKCα, PKCη, and PKCε, did not affect the proliferation but decreased the clonogenicity of the K562 cells by 30% (Figure 1B). On the other hand, 1 μM rottlerin (ROTL) resulted in a 70% inhibition of proliferation and 30% inhibition of clonogenicity, while 5 μM inhibited both by more than 90%. However, because ROTL has also been described to target the mitochondrial respiratory chain [27], we selected the more-specific PKCδ inhibitor, BJE6 [22]. BJE6 dose-dependently inhibited proliferation and clonogenicity and was more powerful than ROTL, resulting in 90% inhibition at 0.3 μM. (Figure 1B,C).

Neither ROTL nor BJE6 affected the expression of PKCδ or its phosphorylation on threonine 505 (Figure S1).

**Figure 2:**
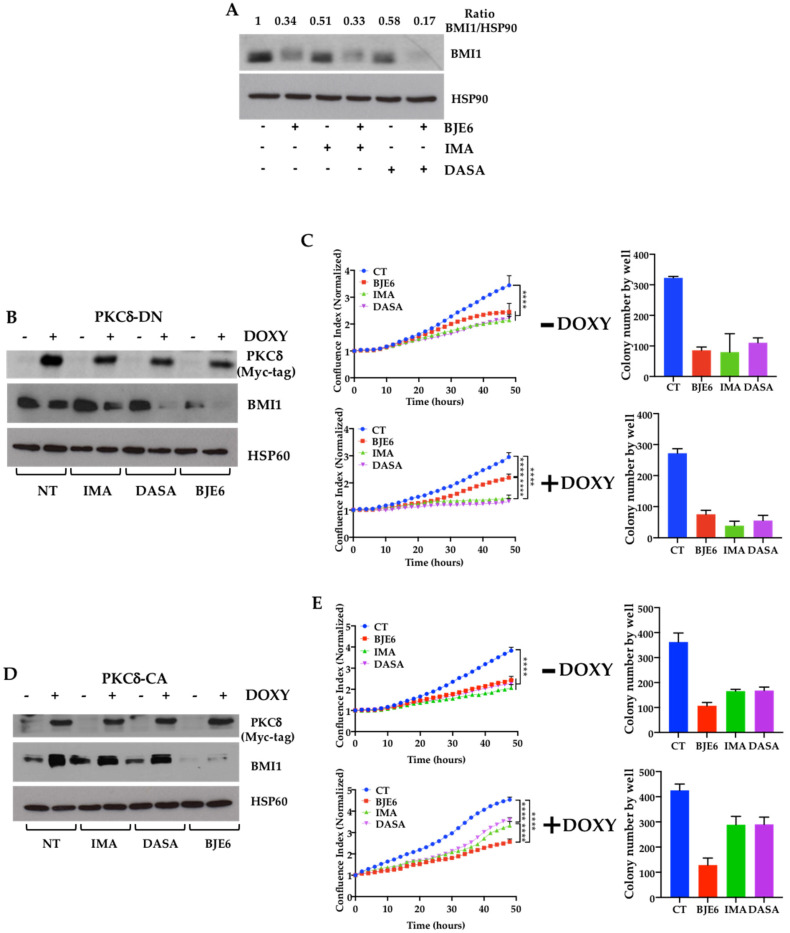
Expression of PKCδ correlated with BMI1 expression in CML cells and controlled proliferation and clonogenicity. (**A**) K562 cells were stimulated or not with BJE6 (0.5 µM), IMA (1 µM), DASA (2 nM), or a combination of BJE6 with any TKI. After 24 h, cell lysates were analyzed by BMI1 immunoblotting. (**B**–**E**) A1-K562 was stimulated with doxycycline (DOXY) (1 µg/mL) for 2 days for induction of DN-PKCδ (**B**,**C**) or CA-PKCδ (**D**,**E**). Cell lysates were analyzed by BMI1 and myc-Tag immunoblotting (**B**,**D**). After DN-PKCδ and CA-PKCδ induction, cells were treated for 48 h (proliferation: (**C**,**E**) left panels) or 7 days (clonogenicity: (**C**,**E**) right panels) with DMSO (CT), 0.1 μM BJE6, 1 μM IMA, or 2 nM DASA. Time-lapse analysis of cells was performed with IncuCyte system. Graphs show quantification of cell numbers from phase-contrast confluence. Data represent mean ± SD (*n* = 3). **** *p* < 0.0001, two-way ANOVA, Tukey’s multiple comparison. A.U., arbitrary units.

We then inhibited PKCδ expression by using siRNA. The downregulation of PKCδ appeared sufficient to inhibit both the expression of BMI1 and the clonogenicity of K562 cells (Figure 1E,F and Figure S2). We then examined whether the downregulation of PKCδ expression could affect the susceptibility of K562 cells to TKIs. We first observed that the decrease in BMI1 expression induced by imatinib (IMA) and dasatinib (DASA) was greater in the si-PKCδ-K562 cells (Figure 1D). A scramble-control siRNA did not modify the sensitivity of the K562 cells to IMA or DASA (Figure 1E,F, upper panels), while the effect of the two TKIs on si-PKCδ-treated K562 cells was more pronounced (Figure 1E,F, lower panels). To confirm that acting on PKCδ could improve the effects of TKIs, we then used A1-K562 cells expressing constitutively active (CA) or dominant negative (DN) PKCδ in a doxycycline (DOXY)-inducible manner. We first observed that the expression of PKCδ-DN decreased BMI1 expression (Figure 2B and Figure S3). Furthermore, PKCδ-DN induction combined with IMA or DASA led to a drop in BMI1 expression, which was slightly enhanced by IMA and more strongly enhanced by DASA and BJE6 (Figure 2B). This paralleled a decrease in both proliferation and clonogenicity (Figure 2C). On the contrary, the ectopic expression of the constitutively active (CA) isoform of PKCδ was associated with higher levels of BMI1 that neither IMA nor DASA was able to reduce, in contrast to BJE6, which decreased both constitutive and PKCδ-CA-induced BMI1 levels (Figure 2D). Furthermore, PKCδ-CA expression appeared to counteract the effects of the two TKIs on cell proliferation and clonogenicity (Figure 2E).

### 2.2. PKCδ Inhibition Synergizes with TKIs to Decrease Proliferation and Clonogenicity of CML Cells

**Figure 3:**
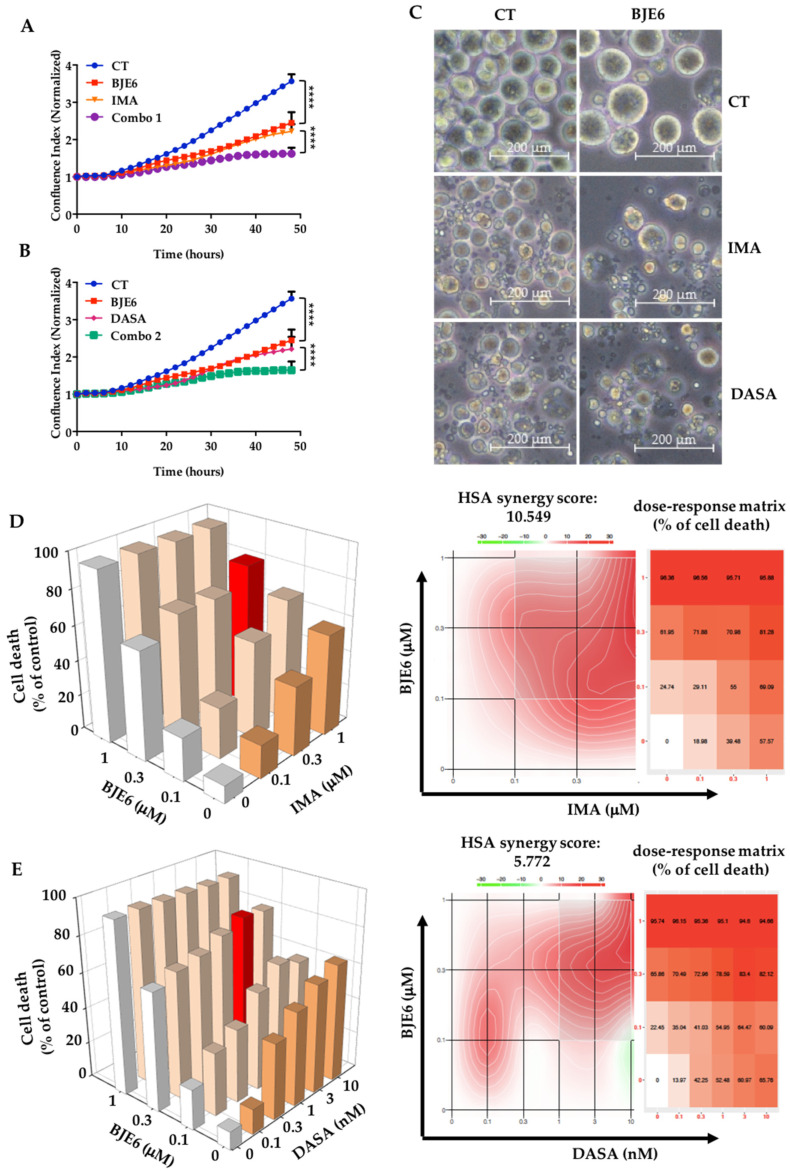
Targeting PKCδ synergized with TKIs to inhibit proliferation and induce cell death of K562 cells. (**A**,**B**) Proliferation curves for K562 cells treated with DMSO (CT), 0.1 µM BJE6, 1 µM IMA, BJE6 and IMA combined (Combo 1), or BJE6 and DASA combined (Combo 2) for 48 h. Time-lapse analysis of K562 cells by phase-contrast microscopy using IncuCyte system. Graphs show quantification of cell numbers from phase-contrast confluence. Data represent mean ± SD (*n* = 3). **** *p* < 0.0001, 2-way ANOVA. A.U., arbitrary units. (**C**) Observation of K562 cells stimulated as in (**A**) and (**B**) by phase-contrast microscopy (Zeiss Axiocam 305 color) at 20×. Scale bar is 200 nm. (**D**,**E**) 3D graphs (left panel) of cell death of K562 cells treated with indicated doses of BJE6, IMA, or DASA according to 4′,6-diamidino-2-phenylindole (DAPI) staining. Cell death is expressed as percentage of control. Best synergistic combinations inducing up to 80% cell death, represented by red histograms, were used for all other experiments. Synergy density plot (right panel) displaying distribution of synergy and dose–response matrix of K562 cells after BJE6/TKI treatment. Combination scores represented by color gradient from green (antagonism) to red (strong synergy). On the dose–response matrix, % cell death represented by color gradient from low to high.

We then examined the simultaneous effect of PKCδ inhibition and BCR-ABL targeting on K562 cells. We observed that 0.1 μM BJE6 combined with 1 μM IMA inhibited K562 cell proliferation by 70%, compared to the 50% inhibition by each drug alone (Figure 3A). Similar results were obtained with DASA (Figure 3B).

Phase-contrast microscopy analysis showed that BJE6 (0.1 μM), IMA (1 μM), DASA (2 nM), or combinations of them decreased cell numbers, with the formation of apparent apoptotic vacuoles at 24 h (Figure S4) and maximal effects at 48 h (Figure 3C). Adding BJE6 to IMA or DASA increased the effects.

In order to characterize the possible effects of the combined inhibition of PKCδ and BCR-ABL, we calculated the synergy score for each dose combination using the SynergyFinder software and the highest single agent (HSA) synergy score, which represents the mean of all the synergy scores. The majority of the combinations for IMA/BJE6 produced highly synergistic results, with an HSA synergy score of 10.549 (Figure 3D). Calculating the individual synergy scores allowed us to identify the best combinations as 0.3 μM BJE6 + 1 μM IMA (the red columns in the histograms of Figure 3D) and 0.1 μM BJE6 + 0.3 μM IMA, leading to 81 and 55% cell death, respectively (Table 1). Concerning the combinations of DASA and BJE6, we observed a lower HSA synergy score of only 5.772, with two distinct zones of very high synergy (Figure 3E). Furthermore, calculating the individual synergy scores indicated that the best combination, inducing up to 80% cell death, was 0.3 μM BJE6 + 3 nM DASA (Table 2), visualized as red columns in the histograms (Figure 3E). We also visualized a second interesting combination with very low concentrations of drugs (0.1 nM DASA + 0.1μM BJE6), which led to only 35% cell death.

### 2.3. PKCδ Inhibition Synergistically Enhances TKI-Induced Mitochondrial Apoptotic Events

**Figure 4:**
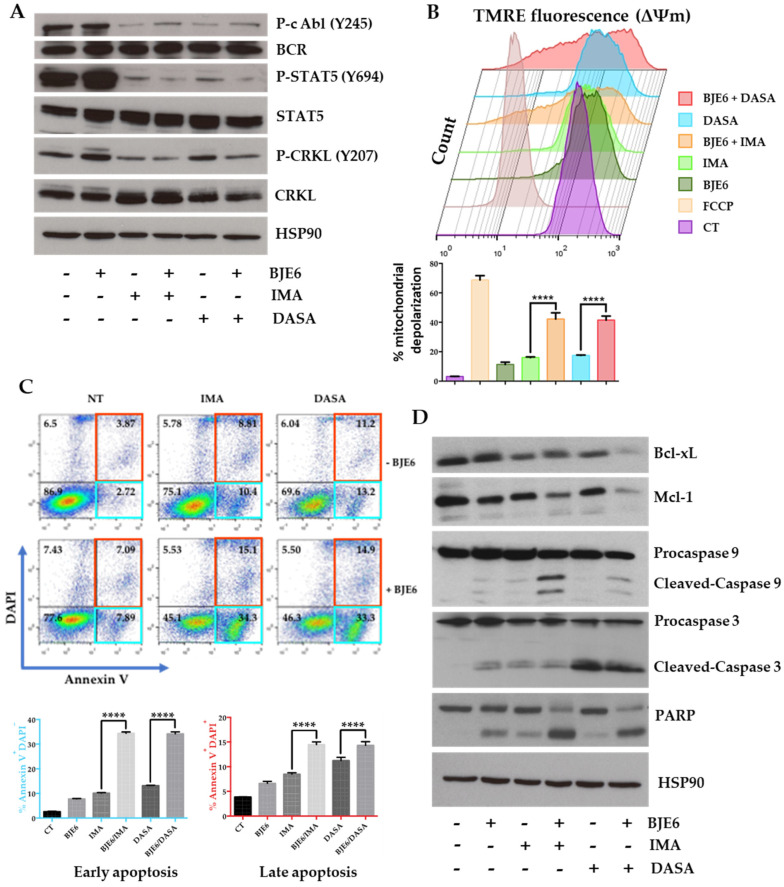
Combinations of PKCδ and BCR-ABL inhibitors induced apoptosis events in K562 cells. (**A**) K562 cells were stimulated or not with 0.5 µM BJE6, 1 µM IMA, 2 nM DASA, or combinations for 15 min. Cell lysates were analyzed by immunoblotting for indicated proteins. (**B**) K562 cells were stimulated or not with 0.5 µM BJE6, 1 µM IMA, 2 nM DASA, or combinations for 24 h. After 15 min of tetramethylrhodamine ethyl ester perchlorate (TMRE) staining (200 nM), 10^5^ cells were analyzed by flow cytometry (upper panel). Quantification of mitochondrial depolarization expressed as % of cells that lost ΔΨm. **** *p* < 0.0001, 2-way ANOVA (lower panel). (**C**) K562 cells were stimulated or not with 0.5 µM BJE6 in combination or not with 1 µM IMA or 2 nM DASA for 24 h. Cytometric analysis after Annexin V/DAPI labelling (left panel). Histograms for Annexin V + DAPI + cells (top-right panel) and Annexin V + DAPI-cells (bottom-right panel). **** *p* < 0.0001, 2-way ANOVA. (**D**) K562 cells were stimulated or not with 0.5 µM BJE6, 1 µM IMA, 2 nM DASA, or combinations for 24 h. Cell lysates were analyzed by immunoblotting for indicated proteins.

In order to understand how PKCδ inhibition altered cell survival, particularly through its synergistic effect with the inhibition of BCR-ABL, we first compared the effects of IMA, DASA, and BJE6 at the level of BCR-ABL and its direct targets: STAT-5 and Crk-like protein (CRKL). We observed that IMA and DASA alone promptly inhibited the phosphorylation of BCR-ABL, STAT5, and CRKL (Figure 4A). BJE6 had no effect by itself and did not significantly influence the effect of each TKI (Figure 4A). Similar results were obtained with the LAMA-84 CML cell line (Figure S5).

Because PKCδ inhibition seemed to induce apoptosis in CML cells (Figure 2C), we next assessed the consequences of this inhibition at the mitochondrial level.

The treatment of K562 cells with BJE6, IMA, or DASA alone for 24 h decreased their mitochondrial membrane potential (MMP) by 16, 18, and 20%, respectively, compared to the mitochondrial uncoupler FCCP (70%) (Figure 4B). Furthermore, the combination of BJE6 with IMA or DASA accentuated the MMP decrease by 2.6- and 2.37-fold, respectively (Figure 4B). Similar results were obtained for LAMA-84 cells. After 24 h, BJE6 enhanced mitochondrial depolarization by 4.6- and 4.4-fold for IMA and DASA, respectively (Figure S6A). After 48 h, BJE6 alone decreased the MMP by 20%, and combination treatment with any TKI enhanced it by up to 80% (Figure S7). Similar results were obtained for the other CML cell line, LAMA-84 (Figure S6B).

We next investigated molecular apoptotic events under the same conditions by flow cytometry following Annexin V/4’, 6-diamidino-2-phenylindole (DAPI) staining, and Western blot analysis. Adding BJE6, IMA, or DASA alone to K562 cells for 24 h was associated with a slight increase in early apoptotic events (Annexin V-positive cells) (Figure 4C) and slight cleavage of caspase 9, caspase 3, and PARP, associated with a small decrease in the expression of the antiapoptotic proteins Bcl-xL and Mcl-1 (Figure 4D). All of these events were greatly amplified when BJE6 was combined with IMA or DASA. BJE6 synergized for early apoptotic events at 24 h (Figure 4C) and late apoptotic events at 48 h (Figure S8). The expression of Bcl-2, another antiapoptotic protein expressed in LAMA-84 cells, behaved in the same way (Figure S9). The inhibition of PKCδ combined with the other available TKIs—nilotinib (NILO), bosutinib (BOSU), and ponatinib (PONA)—led to the same effects on K562 and LAMA-84 cells (Figures S10 and S11).

### 2.4. Combination of BCR-ABL and PKCδ Inhibitors Targeted Stem/Progenitor CML Cells

**Figure 5:**
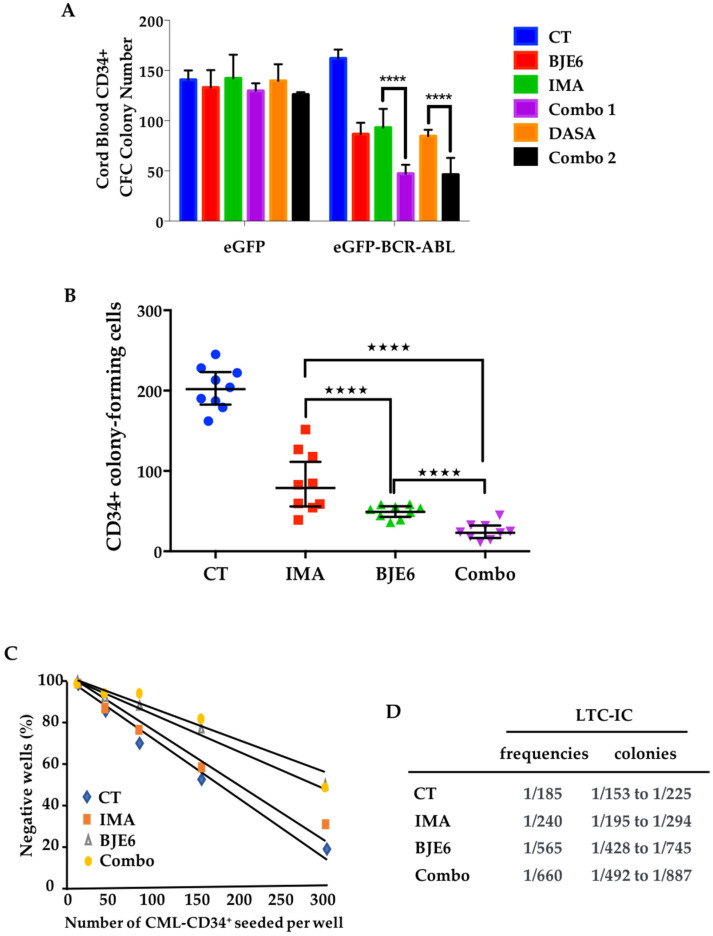
PKCδ inhibition affects CML stem cells. (**A**) Cord blood CD34^+^ cells were transduced with a lentiviral vector expressing BCR-ABL (eGFP-BCR-ABL) or not (eGFP) and incubated for 48 h with DMSO (CT), 0.1 µM BJE6, 1 µM IMA, 2 nM DASA, or a combination of BJE6 and IMA (Combo 1) or BJE6 and DASA (Combo 2). Cells’ clonogenic capacity was examined after 14 days. Error bars represent SD for 3 biological replicates. **** *p* < 0.0001, 2-way ANOVA. (**B**) Clonogenic capacity of primary CD34^+^ cells from 9 diagnosed CML patients, treated for 48 h with DMSO (CT), 0.1 µM BJE6 alone, 1 µg/mL of IMA, or combination of both (Combo). **** *p* < 0.0001, 2-way ANOVA. (**C**) Limited dilution analysis (LDA) of CML LSCs exposed to indicated conditions by long-term culture-initiating cell (LTC-IC) assay. (**D**) LTC-IC frequencies calculated from (**C**).

Before addressing the role of PKCδ in primary CML cells, we sought to identify the maximum concentration of BJE6 that did not affect the differentiation of CD34^+^ cells from healthy donors. BJE6 was well tolerated up to 0.5 μM, while 1 μM BJE6 inhibited differentiation by 50% (Figure S12). We next chose to use BJE6 at 0.1 μM. Healthy CD34^+^ cells from cord blood transduced with *BCR-ABL* produced slightly more colony-forming cell (CFC) colonies than their normal counterparts (Figure 5A). The treatment of these BCR-ABL^+^ cells with 0.1 μM BJE6, 1 μM IMA, or 2 nM DASA clearly decreased CFC colony numbers by 50% but had no effect on normal CD34^+^ cells (Figure 5A). Furthermore, the combined targeting of BCR-ABL and PKCδ inhibited the clonogenic capacity of BCR-ABL-transduced cells by more than 70% without modifying that of mock-transduced cells.

The safety of using these combinations of inhibitors for healthy cells in light of their high efficacy in the oncogenic context led us to examine their effects on primary cells from CML patients. We observed that the inhibition of PKCδ was sufficient to decrease CML CD34^+^ cell clonogenicity by 75%, with a very homogeneous response among patients compared to that realized with IMA, which reduced clonogenicity by only 60% and showed more heterogeneous responses (Figure 5B). The double inhibition of BCR-ABL and PKCδ decreased the clonogenic capacity of CML CD34^+^ cells by 90% using IMA (Figure 5B) or DASA, NILO, BOSU, and PONA (Figure S13), again with homogeneous responses.

We next addressed the role of PKCδ in the maintenance of primitive leukemia cells and evaluated the effect of targeting this kinase alone and in combination with TKIs by carrying out a long-term culture-initiating cell (LTC-IC) assay followed by a limiting dilution assay (LDA). Very importantly, the frequency of untreated CML CD34^+^ cells (1/185) measured by LTC-IC/LDA was only slightly reduced by IMA (1/240), while BJE6 alone was very effective at significantly reducing their frequency, by 3.1-fold (1/565) (Figure 5C,D). Furthermore, combining the two drugs further improved the effect, with a 3.56-fold decrease in frequency (1/660) (Figure 5C,D).

## 3. Discussion

We show here that the pharmacological targeting of PKCδ in CML-CD34^+^ cells decreased LTC-IC frequency, demonstrating that this treatment can affect the pool of CML progenitors/LSCs in vitro. We also demonstrated that the inhibition of PKCδ could synergize with any of the three generations of TKIs to potentiate CML cell death and decrease the clonogenic potential of CML-CD34^+^ cells. Our results are in line with a previously observed supporting role of PKCδ in cancer stem cells (CSCs). The inhibition of PKCδ has been demonstrated to eliminate CSCs in different solid tumors, such as melanoma [28], pancreatic cancer [22,29], liver cancer [30], and lung cancer [23]. Furthermore, the fact that inhibiting PKCδ did not affect the proliferation or survival of normal cells favors the idea of some tumor-specificity of a PKCδ-targeted approach [22].

We observed that PKCδ inhibition decreased BMI1 expression, associated with a loss of CML cell clonogenicity. BMI1, a polycomb protein and component of polycomb repressive complex 1 (PRC1), is crucial for self-renewal. It regulates oncogenic phenotypes and promotes CSCs and therapy resistance in cancer cells [31,32]. Pharmacological BMI1 inhibitors such as PTC-209 [33,34,35] and PTC-296 [36] have shown promising results in different types of cancers, but associated treatments have not yet been offered. Our results indicate that the decrease in BMI1 expression triggered by PKCδ inhibition could be an important feature of PKCδ inhibitors and explain their effectiveness.

It has recently been shown that the PPARγ agonists glitazones could decrease the expression of STAT5, a substrate crucial for BCR-ABL oncogenic signaling. This led to a strong decrease in the CML-LSC pool, and three patients under imatinib treatment who were given pioglitazone showed sustained, complete molecular responses [15]. We show that PKCδ inhibition did not affect the BCR-ABL-STAT5-CRKL signaling pathway. Rather, the most pronounced effect of PKCδ inhibition in combination with TKIs was a decrease in the expression of prosurvival molecules of the Bcl2 family: Bcl-2, Bcl-xL, and Mcl-1. This could explain why and how BJE6 can sensitize LSCs to TKIs while sparing HSCs, as Bcl-2 was shown to be overexpressed in quiescent LSCs but not in HSCs [37]. Along this line, pan-Bcl-2 inhibitors were demonstrated to be efficient in sensitizing human bone-marrow LSCs to TKIs [38].

Numerous studies have described the implication of Bcl-2 family members for LSC survival, but the inhibition of Bcl-2 with ABT-737 was reported to be insufficient for preventing disease progression in a mouse lymphoma model [39]. Clearly, there is a need to also inhibit Mcl-1, which is implicated in both leukemogenic acceleration [40] and resistance [41,42,43]. The fact that it may be necessary to block these two prosurvival Bcl-2 family members to initiate apoptosis [42] makes pan-Bcl-2 inhibitors very attractive for cancer therapy. In this context, BJE6, which decreases the expression of both Bcl-2 and Mcl-1, could be an attractive molecule. In addition, it was recently shown in AML that Bcl-2 inhibition enhances the mitochondrial priming toward apoptosis induced by the Src family kinase inhibitor RK-20449 [44]. This could explain the synergistic effect we observed in tetramethylrhodamine ethyl ester perchlorate (TMRE) experiments when we combined BJE6 with the dual BCR-ABL/SRC inhibitor dasatinib (DASA). Nevertheless, PKCδ inhibition combined with DASA has proven to be different in its effects to combinations of BJE6 with any other TKI, notably, with two distinct ranges of concentrations providing very strong synergies. This could be the consequence of both Src inhibition with DASA and the partial control of PKCδ by Src kinases [45]. The combination of DASA and BJE6 was particularly synergistic at very low concentrations of both drugs. This may be of additional interest in the chronic treatment of the pathology due to making it possible to reduce the dosage of the molecules to attenuate the possible side effects. A BJE6/DASA combination may also be of great interest in the context of CML resistance associated with the overexpression of Src kinases.

The only way to cure CML and stop treatment without relapse is, probably, to not only eliminate differentiated mature leukemia cells, but also eradicate leukemia progenitors and LSCs. We propose a dual approach that combines PKCδ and BCR-ABL inhibition as very effective against both proliferative CML cells and progenitors, in association with any TKI available as first line treatment. This combination will have to be tested on TKI-insensitive quiescent CML cells to evaluate whether it is a new opportunity for personalized treatment with high efficacy.

## 4. Materials and Methods

### 4.1. Reagents and Antibodies

RPMI 1640 medium, penicillin, streptomycin, sodium pyruvate, and fetal calf serum were purchased from Gibco (Invitrogen Life Technologies, Waltham, MA, USA). Sodium fluoride, sodium orthovanadate, aprotinin, leupeptin, phenyl-methylsulfonyl fluoride, and Triton X-100 were purchased from Sigma (St. Louis, MO, USA). Imatinib mesylate was purchased from Enzo Life Sciences (Farmingdale, NY, USA); nilotinib, from Selleck Chemicals (Houston, TX, USA); and doxycycline, from Calbiochem (Darmstadt, GERMANY). Validated small-interfering RNAs (siRNAs) were obtained from Thermo Fisher Scientific (Waltham, MA, USA). The antibodies used in these studies are listed in Table 3.

### 4.2. Cell Lines

The human CML cell lines LAMA-84 and K562 (ATCC) and A1-K562 [26] were regularly tested for mycoplasma contamination (MycoAlert Mycoplasma Detection Kit, Lonza, Basel, Switzerland, #LT07-418). A1-K562 cells expressing the tetracycline repressor (Tet-K562) were established by the stable transfection of the pCDNA6-TR plasmid (Invitrogen Life Technologies) as previously described [26]. The mouse M2-10B4 cell line, derived from bone marrow stromal cells, was purchased from STEMCELL Technologies (Vancouver, BC, Canada).

All the cell lines were cultured in RPMI 1640 medium supplemented with 5% fetal calf serum (FCS), 1 mM sodium pyruvate, 50 U/mL penicillin, and 50 μg/mL streptomycin.

### 4.3. Proliferation Assay

Cell proliferation was assessed with IncuCyte technology. Cells were grown and treated on 12-well plates and analyzed on an IncuCyte ZOOM imaging system (Essen Bioscience, Ann Arbor, MI, USA). The optical density of the cells was measured in 9 squares per pit (in triplicate), with 4 × 10^5^ cells seeded per pit, and real-time proliferation was assessed from Hours 1 to 48.

### 4.4. Viability Assay and Synergy Score Calculation

Cells were stained with DAPI prior to flow cytometric analysis in 96-well plates and analyzed with a MACSQuant or MACSQuant VYB flow cytometer (Miltenyi Biotec, Bergish Gladbach, Germany). DAPI-negative cells were counted in terms of absolute number, and those for the treated conditions were normalized to those for the control.

The calculation of the synergy scores was performed with the SynergyFinder software (https://synergyfinder.fimm.fi, accessed on 10 October 2020). Briefly, the synergy scores for the drug combinations were averaged over all the dose combination measurements. The highest single agent (HSA) model was used to quantify the degree of synergy. Dose–response curves were fitted with a 4-parameter logistic regression (LL4), and readout viability baseline correction was applied.

### 4.5. Analysis of Apoptosis

Apoptosis was assessed using Annexin V-DAPI staining. Briefly, cells were washed with phosphate-buffered saline (PBS) with 2% FCS, and then stained in a buffer containing FITC Annexin V (Miltenyi Biotec, 130-097-928) and DAPI (Sigma, D9542-5MG) for 15 min at room temperature (°C) according to the manufacturer’s protocol. The cells were washed, resuspended in PBS with 2% FCS, and analyzed by flow cytometry (MACSQUANT 10). The FlowJo^®^ v10 software was used to analyze the data.

### 4.6. Mitochondrial Membrane Potential (ΔΨm) Measurement

The ΔΨm was measured after tetramethylrhodamine ethyl ester perchlorate (TMRE) incorporation by flow cytometry (Sigma, T669). TMRE is a cell permeant and positively charged dye that readily accumulates in active mitochondria due to their relative negative charge. Damaged and/or inactive mitochondria have a loss of membrane potential, resulting in decreased TMRE incorporation. Cells were washed with PBS with 2% FCS and then stained (10^5^ cells) with 200 nM TMRE for 15 min at room temperature (20 °C). The cells were washed, resuspended in PBS with 2% FCS, and analyzed by flow cytometry (MACSQUANT 10). The FlowJo^®^ v10 software was used to analyze the data.

### 4.7. Primary Cell Isolation

Bone marrow samples were collected from patients newly diagnosed with CML as part of an institutionally approved protocol for cell sample collection (Centre Hospitalier Nice, 06204 Nice, France). Cord blood and bone marrow samples were collected from healthy donors with informed consent. Mononuclear cells were isolated by density centrifugation (Ficoll-Paque Plus, STEMCELL Technologies), washed with PBS with 5% fetal calf serum (FCS). For clonogenic assays CD34^+^ cells were positively enriched with CD34 microbeads (CD34 MicroBead Kit, Miltenyi Biotec). For LTC-IC, CD34^+^ cells were first positively enriched and then depleted for CD38^+^ cells (human CD34^+^CD38^−^ isolation kit, Miltenyi Biotec). The CD34^+^ cells, after sorting, were stained with anti-human-CD34 antibody (Miltenyi Biotec, 130-113-741) and checked by flow cytometry to ensure purity (always above 96%).

### 4.8. Colony-Forming Cell (CFC) Assay

Cells from human cell lines were grown on 12-well plates (3 × 10^3^ cells/well) on semisolid methyl cellulose medium (STEM MACSQ HCF-CFU Basic, Miltenyi Biotec) in the presence of the indicated drugs. After 7 days, colonies were labelled with MTT and quantified with the ImageJ software.

Primary CD34^+^ CML cells were plated in serum-free medium (HPC Expansion medium, PromoCell) in the presence of the indicated drugs. After 48 h, cells (0.9 × 10^3^) from each condition were transferred into 1 mL of alpha-MEM-based methylcellulose medium (GF H4434, STEMCELL Technologies) in 35 mm tissue culture dishes. The cells were scored and collected after 14 days of incubation at 37 °C and 5% CO_2_.

### 4.9. Long-Term Culture-Initiating Cell (LTC-IC) Experiments with Limiting Dilution Assay (LDA)

LTC-IC experiments with the LDA were performed in StemSpan SFEM medium (STEMCELL Technologies) on irradiated stromal M2.10B4 monolayers with several dilutions of CD34^+^ cells (300, 150, 75, or 37 cells per well) in 96-well plates with 16 replicate wells per concentration. The cells were kept for a minimum of 5 weeks with a half-medium change once per week. After this period, all the remaining cells from each LTC were assessed by CFC in alpha-MEM-based methylcellulose medium (GF H4434, STEMCELL Technologies), and the frequencies were calculated with the L-Calc software.

### 4.10. Plasmid, Transfection and Luciferase Reporter Assays

All the PKC constructs were cloned into the pcDNA4 vector (Invitrogen Life Technologies). Tet-K562 cells were transfected with 1 µg of each PKC-containing vector using the Nucleofactor Amaxa protocol T16 and kit V (LonzaAG) to create corresponding Tet-On-inducible PKC.

BCR-ABL lentiviral vector: The amplification product was subcloned into a pCR(R)-XL TOPOR plasmid (Invitrogen Life Technologies) before being inserted into the SIV-GAE-SSFV lentiviral transfer vector, followed by DNA sequencing. An SIV-GAE-SSFV eGFP vector was used as a control. The SIV vectors were produced as previously described [46].

The lentiviral transduction of CD34^+^ cells: Cells were suspended (5 × 10^4^/mL) in StemSpan medium (STEMCELL Technologies) supplemented with protamine sulfate (4 µg/mL), stem cell factor (SCF) (100 ng/mL), Flt-3-L (100 ng/mL), IL-3 (20 ng/mL), and IL-6 (20 ng/mL) in a 96-well plate coated with RetroNectinR (Takara Shuzo Co, Kyoto, Japan). The cell suspensions were incubated for 16 h. Lentiviral vectors were then added to the cell suspensions for a 12-h incubation period. The cells were washed twice before seeding. The transduction efficiency was evaluated at Day 3 by GFP detection using flow cytometry. For the luciferase assays, 10^6^ cells were plated in 24-well plates, and the cells were electroporated with the Amaxa Cell Line Nucleofector Kit V with 1 μg of respective reporter plasmid and other plasmids. Then, 16 h after transfection, the cells were lysed with 80 μL of lysis buffer (Promega, Madison, WI, USA) for 20 min. The luciferase activity of 20 μL of cell lysate was measured using the dual luciferase reporter assay system.

### 4.11. Western Blotting

Cell extracts were made in lysis buffer (50 mM HEPES at pH 7.4, 150 mM NaCl, 20 mM EDTA, 100 µM NaF, 10 mM Na_3_VO_4_, 1% Nonidet P-40, and protease inhibitors). Proteins (40 µg) were separated on 10% SDS-PAGE gels. Western blotting was performed using standard methods, and ECL detection (Amersham Pharmacia, Chicago, Illinois, USA) and light signals were recorded as previously described [26]. For all experiments, uncropped Western blot images are presented in Figure S14.

### 4.12. Stastitical Analysis

All the statistical analyses were performed using Prism 6 (GraphPad Software, San Diego, CA, USA). For studies involving 2 independent groups, t-tests were performed. For 3 or more independent groups, 2-way ANOVA with Tukey’s post hoc test was performed. All the tests were 2-tailed. Throughout, asterisks indicate significance (* *p* < 0.05; ** *p* < 0.01; *** *p* < 0.001; **** *p* < 0.0001).

## 5. Conclusions

To the best of our knowledge, the present study is the first to determine the biological role of PKCδ in both the proliferation of differentiated CML cells and the self-renewal of CD34^+^ progenitor/leukemic stem cells. The targeting of PKCδ in combination with any of the first-, second-, and third-generation TKIs could represent new options for targeting the leukemic stem cell compartment at the root of resistance and disease relapse.

## Figures and Tables

**Table 1:** Relative synergy of BJE6 and IMA for K562 cells calculated from data obtained and shown in Figure 2D. Best relative synergy in bold.

BJE6 (µM)	IMA (µM)	% Cell Death	Relative Synergy	Standard Deviation
0	0	0	0	0
0.1	0	24.74	0	12.23
0.3	0	61.95	0	0.56
1	0	96.36	0	0.61
0	0.1	18.98	0	9.98
0.1	0.1	29.11	5.325283	0.73
0.3	0.1	71.88	12.1007	8.41
1	0.1	96.56	0.24372	0.39
0	0.3	39.48	0	3.64
**0.1**	**0.3**	**55**	**18.91267**	**4.63**
0.3	0.3	70.98	11.00396	1.84
1	0.3	95.71	−0.79209	0.59
0	1	57.57	0	2.55
**0.1**	**1**	**69.09**	**14.03827**	**1.36**
**0.3**	**1**	**81.28**	**23.55554**	**0.47**
1	1	95.88	−0.58493	0.18

**Table 2:** Relative synergy of BJE6 and DASA for K562 cells calculated from data obtained in Figure 2E. Best relative synergy in bold.

BJE6 (µM)	DASA (nM)	% Cell Death	Relative Synergy	Standard Deviation
0	0	0	0	0
0.1	0	22.45	0	7.18
0.3	0	65.86	0	4.01
1	0	95.74	0	0.55
0	0.1	13.97	0	7.27
**0.1**	**0.1**	**35.04**	**14.92119**	**5.59**
0.3	0.1	70.49	5.487299	0.86
1	0.1	96.15	0.485916	0.60
0	0.3	42.25	0	4.11
0.1	0.3	41.03	−1.4459	2.99
0.3	0.3	72.96	8.414648	2.29
1	0.3	95.36	−0.45036	0.68
0	1	52.48	0	10.10
0.1	1	54.95	2.92735	5.51
**0.3**	**1**	**78.59**	**15.08711**	**4.65**
1	1	95.1	−0.7585	1.13
0	3	60.97	0	8.02
0.1	3	64.47	4.148066	4.41
**0.3**	**3**	**83.4**	**20.78774**	**4.20**
1	3	94.6	−1.35108	0.89
0	10	65.76	0	6.63
0.1	10	60.09	−6.71987	2.80
0.3	10	82.12	19.27073	2.25
1	10	94.66	−1.27997	0.59

**Table 3:** Antibodies used in Western blotting experiments.

Target	Antibody	Ref	Supplier
Actin	Actin (I-19)	sc-1616	SCB
Bcl-2	Bcl-2 (C 21)	sc-783	SCB
Bcl-xL	Bcl-xL	2762	CST
Bmi1	Bmi1 (F6)	05-637	Merck Millipore (Upstate)
BCR	BCR	3902 S	CST
Caspase 3	Caspase 3	9662 S	CST
Caspase 9	Caspase 9	9502 T	CST
CRKL	CRKL (32H4)	3182	CST
HSP60	HSP60 (K-19)	sc-1722	SCB
HSP90	HSP 90α/β Antibody (F-8)	sc-13119	SCB
Mcl-1	Mcl-1 (D35A5)	5453 S	CST
Myc-tag	Myc-tag clone 4A6	05-724	Merck Millipore (Upstate)
PARP	PARP	9542 S	CST
P-c Abl	P-c Abl (Y245) (73E5)	2868 S	CST
P-CRKL	P-CRKL (Tyr207)	3181 S	CST
P-STAT5	P-STAT5 (Tyr694) (C11C5)	9359 S	CST
PKCδ	PKCδ	2058 S	CST
P-KCδ	P-PKCδ (Thr505)	9374S	CST
STAT5	STAT5 (D2O6Y)	94205	CST

## Data Availability

Data is contained within the article or supplementary material.

## Supplementary Materials

The following are available online at https://www.mdpi.com/article/10.3390/cancers13071693/s1. Figure S1. Effect of PKC inhibitors on BMI1 and PKCδ expression, Figure S2. PKCδ controlled BMI1 transcription, Figure S3. Induction of PKCδ-DN decreased BMI1 expression, Figure S4. Dual inhibition of PKCδ and BCR-ABL enhanced K562 cell death, Figure S5. Inhibition of PKCδ did not affect BCR-ABL-STAT5-CRKL pathway. Figure S6. Inhibition of PKCδ enhanced mitochondrial depolarization induced by TKIs in LAMA-84 cells, Figure S7. Inhibition of PKCδ enhanced mitochondrial depolarization induced by TKIs in K562 cells, Figure S8. Inhibition of PKCδ enhanced late apoptosis induced by TKIs in K562 cells, Figure S9. Inhibition of PKCδ enhanced biochemical apoptotic events induced by TKIs in CML cells, Figure S10. Inhibition of PKCδ enhanced early apoptosis induced by TKIs in CML cells, Figure S11. Inhibition of PKCδ enhanced apoptosis induced by any TKI in CML cells, Figure S12. Evaluation of BJE6 toxicity in healthy CD34^+^ cells, Figure S13. Dual inhibition of PKCδ and BCR-ABL inhibited CML CD34^+^ clonogenicity. Figure S14: Uncropped Western blot images. Table S1. Relative cell number and standard deviation used to calculate percentage of response. Table S1. Relative to Figure 4B, Table S2. Relative to Figure 4C, Table S3. Relative to Figure 4C.

## Abbreviations

Bcl-2	B-cell lymphoma 2
Bcl-xL	B-cell lymphoma–extra large
BCR-ABL	Breakpoint cluster region–Abelson murine leukemia viral oncogene homolog 1
BJE6	BJE6-106
BMI1	B cell-specific Moloney murine leukemia virus integration site 1
BOSU	Bosutinib
CA	Constitutively active
CFC	Colony-forming cell
CRKL	Crk-like protein
CSC	Cancer stem cell
DAPI	4′,6-diamidino-2-phenylindole
DASA	Dasatinib
DN	Dominant negative
DOXY	Doxycycline
EGFR	Epithelial growth factor receptor
ENZA	Enzastaurin
GFX	GF109203X
HSC	Hematopoietic stem cell
IMA	Imatinib
LIC	Leukemia-initiating cell
LSC	Leukemic stem cell
Mcl-1	Induced myeloid leukemia cell differentiation protein
NILO	Nilotinib
PKCδ	Protein kinase Cδ
PONA	Ponatinib
PRC1	Polycomb repressive complex 1
SOTRA	Sotrastaurin
STAT5	Signal transducer and activator of transcription 5
TKI	Tyrosine kinase inhibitor
TMRE	Tetramethylrhodamine ethyl ester perchlorate
