# Supplementary material for: Combination of PKCδ Inhibition with Conventional TKI Treatment to Target CML Models
Source: Cancers (Basel). 2021 Apr 2;13(7):1693. doi: 10.3390/cancers13071693 (PMC8038300; doi:10.3390/cancers13071693)
Supplement: Supplementary file 1 [file cancers-13-01693-s001.zip › cancers-1161399-supplementary-proof/cancers-1161399- Supplementary-proof.docx]

Combination of PKCδ Inhibition with Conventional TKI
Treatment to Target CML Models ^†^

Fabien Muselli, Lucas Mourgues, Rita Morcos, Nathalie Rochet, Marielle Nebout, Agnès Guerci, Douglas V Faller, Rana Mhaidly ^1^, Els Verhoeyen, Laurence Legros, Jean-François Peyron and Didier Mary

**
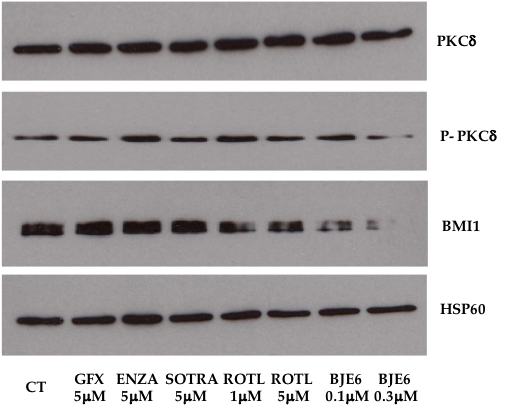
**

**Figure S1.** Effects of PKC inhibitors on BMI1 and PKCδ expression. K562 cells were treated for 24 h with DMSO (CT) or indicated concentrations of enzastaurin (ENZA), sotrastaurin (SOTRA), GF109203X (GFX), rottlerin (ROTL), or BJE6-106 (BJE6). Cell lysates were analyzed by immunoblotting for indicated proteins.


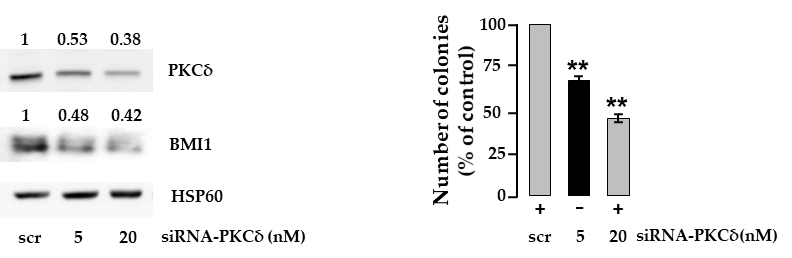


**Figure S2.** PKCδ controls BMI1 transcription. K562 cells transfected with PKCδ siRNA. BMI1 and PKCδ immunoblotting 24 h after transfection (left panel). Clonogenicity potential 7 days after transfection (right panel). Results are expressed as % of control (278 ± 28%).


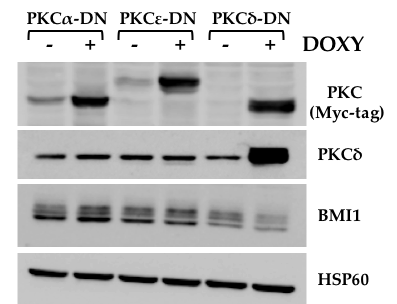


**Figure S3.** Induction of PKCδ-DN decreases BMI1 expression. A1-K562 were stimulated by DOXY (1 µg/mL) for 2 days for indicated DN-PKC induction. Cell lysates were analyzed by BMI1, PKCδ, and myc-Tag immunoblotting.


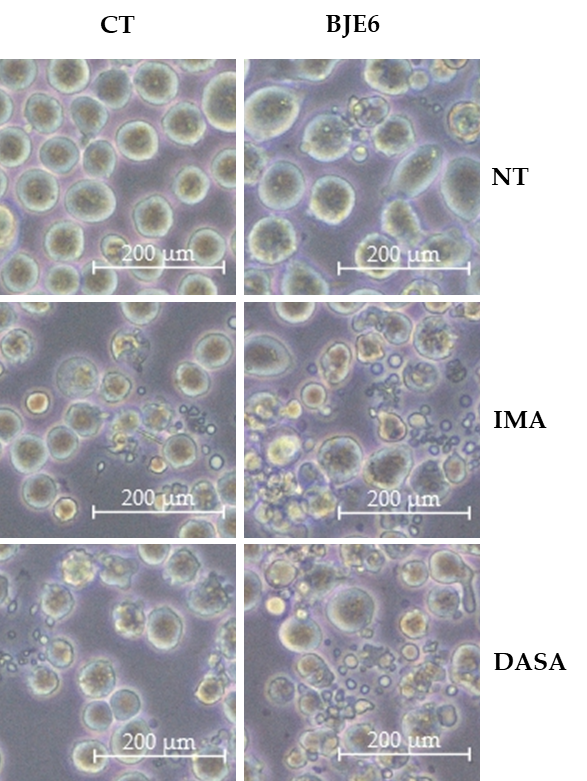


**Figure S4.** Dual inhibition of PKCδ and BCR-ABL enhanced K562 cell death. K562 cells stimulated for 24 h with indicated conditions: with vehicle (CT), 0.5 µM BJE6, 1 µM IMA, 2 nM DASA, or combinations, observed by phase-contrast microscopy (Zeiss Axiocam 305 color) at 20×. Scale bar is 200 µm.

**
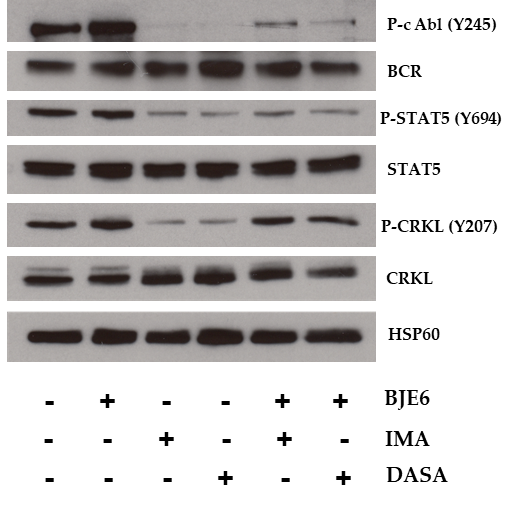
**

**Figure S5.** Inhibition of PKCδ did not affect BCR-ABL–STAT5–CRKL pathway. LAMA-84 cells were stimulated or not with 0.5 µM BJE6, 1 µM IMA, 2 nM DASA, or combinations for 15 minutes. Cell lysates were analyzed by immunoblotting for indicated proteins.


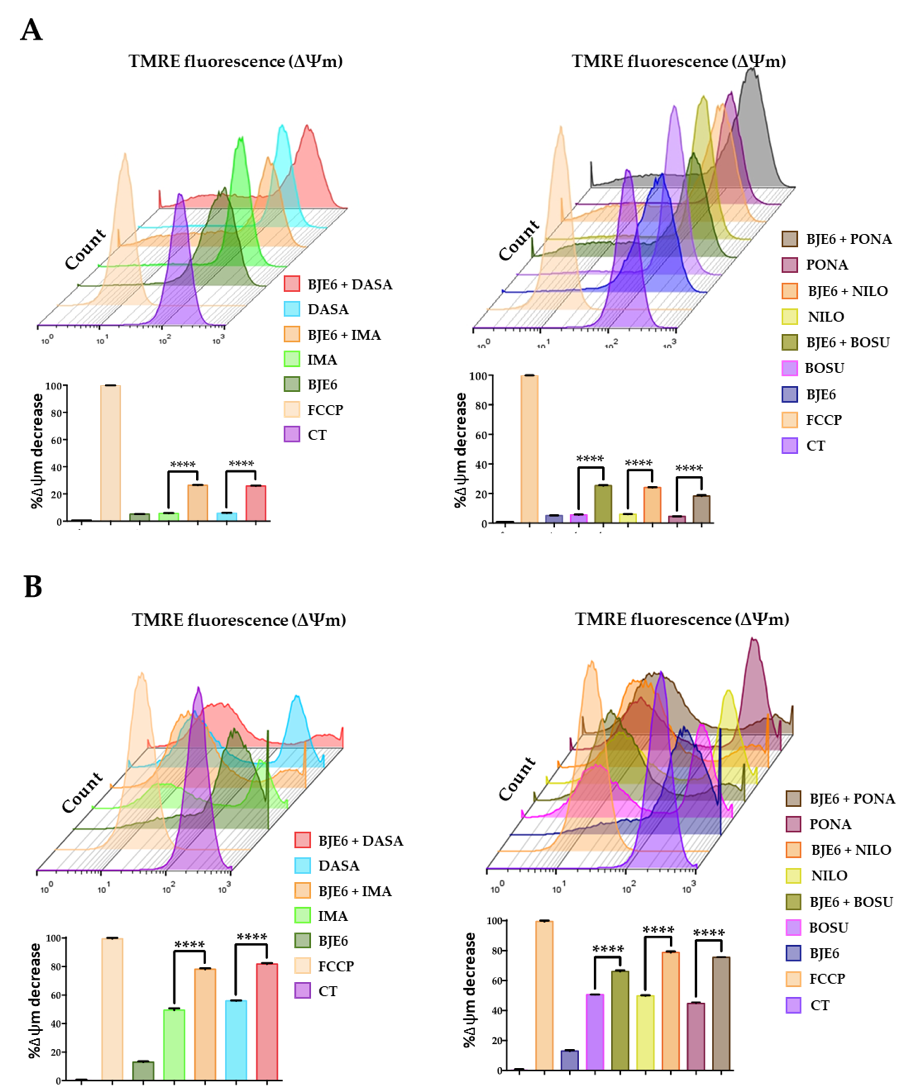


**Figure S6.** Inhibition of PKCδ enhanced mitochondrial depolarization induced by TKIs in LAMA-84 cells. LAMA-84 cells underwent mitochondrial depolarization upon indicated treatments for (**A**) 24 h and (**B**) 48 h. Cytometric analysis of 10^5^ cells after TMRE staining (top panels) and quantification of mitochondrial depolarization expressed as % of cells that lost ΔΨm (bottom panels).

**Table** Relative to Fig S6A

|  | **CT** | **FCCP** | **BJE6** | **IMA** | **BJE6/IMA** | **DASA** | **BJE6/DASA** |
| --- | --- | --- | --- | --- | --- | --- | --- |
| Relative Cell Number | 267.88 | 33462.67 | 1718.94 | 1919.86 | 8829.14 | 1964.51 | 8639.38 |
| Standard Deviation | 0 | 12.12 | 38.66 | 77.33 | 84.27 | 77.33 | 115.99 |

|  | **BOSU** | **BJE6/BOSU** | **NILO** | **BJE6/NILO** | **PONA** | **BJE6/PONA** |
| --- | --- | --- | --- | --- | --- | --- |
| Relative Cell Number | 1819.40 | 8471.95 | 1986.83 | 8058.96 | 1506.87 | 6127.93 |
| Standard Deviation | 84.27 | 133.94 | 77.33 | 84.27 | 33.48 | 219.58 |

**Table** Relative to Fig S6B

|  | **CT** | **FCCP** | **BJE6** | **IMA** | **BJE6/IMA** | **DASA** | **BJE6/DASA** |
| --- | --- | --- | --- | --- | --- | --- | --- |
| Relative Cell Number | 232.08 | 30096.42 | 3935.36 | 14974.54 | 23632.34 | 16932.13 | 24752.40 |
| Standard Deviation | 17.47 | 189.94 | 184.13 | 378.50 | 196.96 | 63.01 | 194.62 |

|  | **BOSU** | **BJE6/BOSU** | **NILO** | **BJE6/NILO** | **PONA** | **BJE6/PONA** |
| --- | --- | --- | --- | --- | --- | --- |
| Relative Cell Number | 15337.81 | 20019.88 | 15095.63 | 23844.24 | 13531.58 | 22835.17 |
| Standard Deviation | 17.47 | 235.13 | 106.31 | 192.25 | 228.54 | 87.38 |


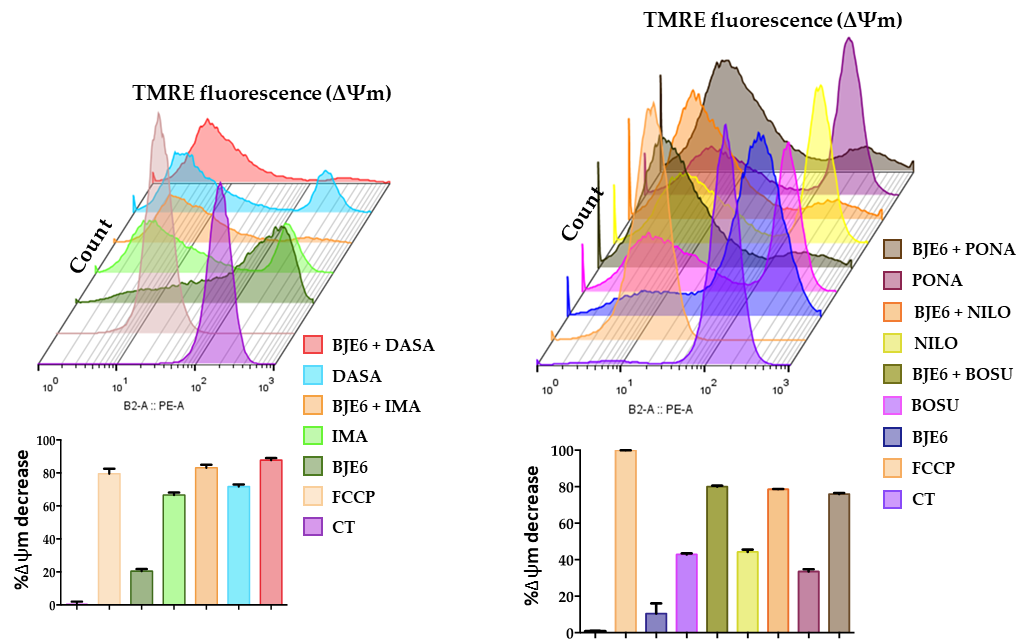


**Figure S7.** Inhibition of PKCδ enhances mitochondrial depolarization induced by TKIs in K562 cells. K562 cells stimulated or not with indicated conditions for 48 h. Cytometric analysis of 10^5^ cells after TMRE staining (top panels) and quantification of mitochondrial depolarization expressed as % of cells that lost ΔΨm (bottom panels).

|  | **CT** | **FCCP** | **BJE6** | **IMA** | **BJE6/IMA** | **DASA** | **BJE6/DASA** |
| --- | --- | --- | --- | --- | --- | --- | --- |
| **Relative Cell Number** | **343.34** | **24814.33** | **6388.26** | **20798.26** | **25948.40** | **22379.72** | **27373.80** |
| **Standard Deviation** | **297.75** | **937.94** | **423.77** | **454.82** | **575.82** | **419.92** | **422.24** |

|  | **CT** | **FCCP** | **BJE6** | **BOSU** | **BJE6/BOSU** | **NILO** | **BJE6/NILO** | **PONA** | **BJE6/PONA** |  | |
| --- | --- | --- | --- | --- | --- | --- | --- | --- | --- | --- | --- |
| **Relative Cell Number** | **270.51** | **31167.01** | **6256.55** | **13390.37** | **24970.40** | **13796.14** | **24512.60** | **10466.75** | **23711.47** |  |  |
| **Standard Deviation** | **18.02** | **35.92** | **174.46** | **173.78** | **165.16** | **409.35** | **47.67** | **387.76** | **171.90** |  |  |


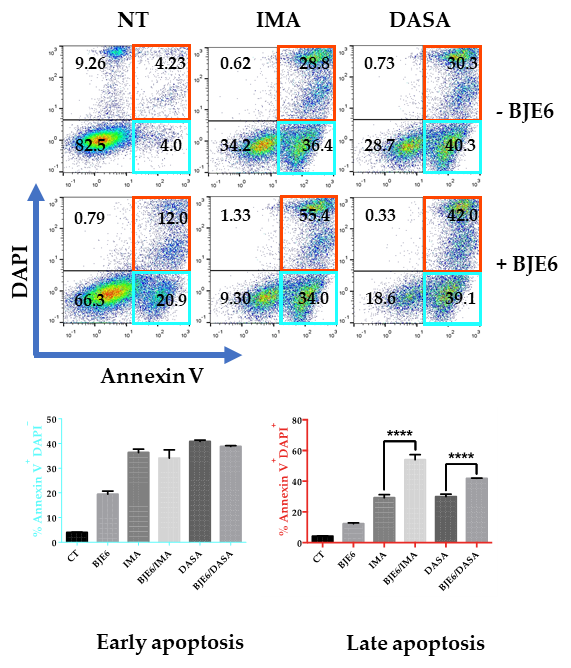


**Figure S8.** Inhibition of PKCδ enhanced late apoptosis induced by TKIs in K562 cells. K562 cells were stimulated or not with 0.5 µM BJE6 in combination or not with 1 µM IMA or 2 nM DASA for 48 h. Cytometric analysis after Annexin V/DAPI labelling (top). Histograms for Annexin V + DAPI – cells (bottom-left panel) and Annexin V + DAPI + cells (bottom-right panel).

early apoptosis

|  | **CT** | **BJE6** | **IMA** | **BJE6/IMA** | **DASA** | **BJE6/DASA** |
| --- | --- | --- | --- | --- | --- | --- |
| Relative Cell Number | 1310.93 | 6401.22 | 11958.45 | 11213.10 | 13438.18 | 12769.56 |
| Standard Deviation | 26.57 | 416.37 | 444.02 | 1101.94 | 168.74 | 100.45 |

late apoptosis

|  | **CT** | **BJE6/E6** | **IMA** | **BJE6/IMA** | **DASA** | **BJE6/DASA** |
| --- | --- | --- | --- | --- | --- | --- |
| Relative Cell Number | 1420.54 | 4099.41 | 9634.71 | 17756.82 | 9875.86 | 13767.01 |
| Standard Deviation | 31.36 | 148.27 | 656.83 | 1094.56 | 531.58 | 50.23 |

**
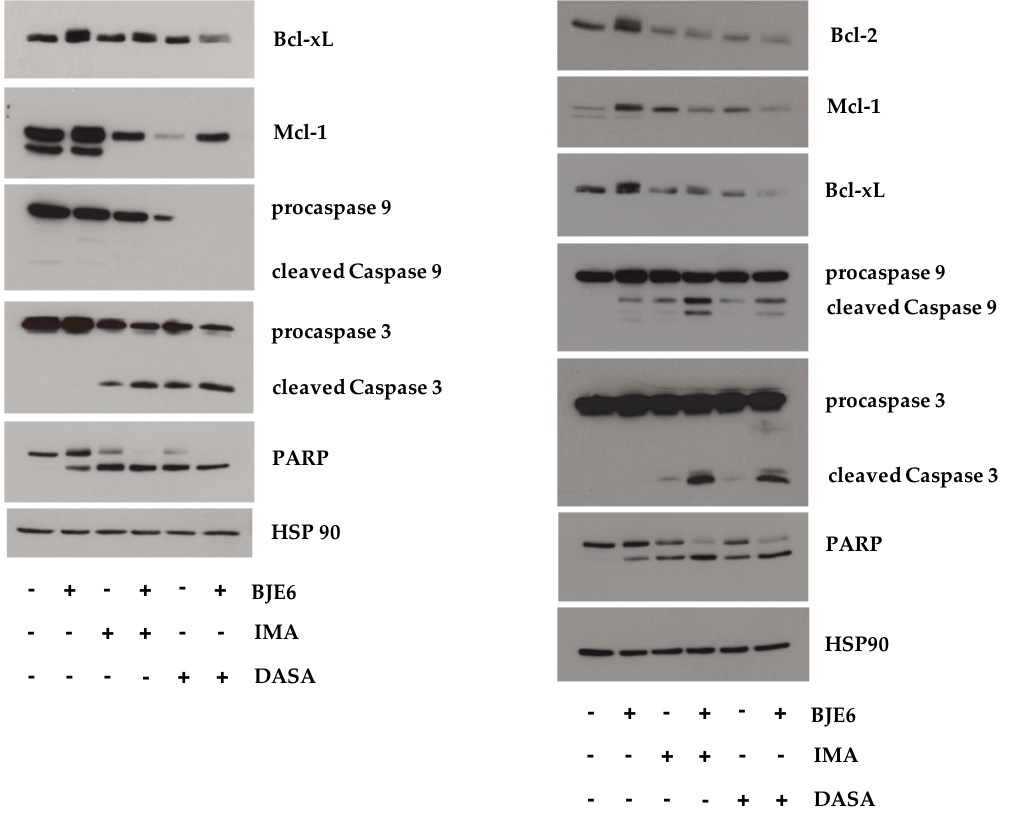
**

**Figure S9.** Inhibition of PKCδ enhanced biochemical apoptotic events induced by TKIs in CML cells. K562 cells (left) and LAMA-84 cells (right) stimulated or not with 0.5 µM BJE6, 1 µM IMA, 2 nM DASA, or combinations for 48 and 24 h, respectively. Cell lysates were analyzed by immunoblotting.


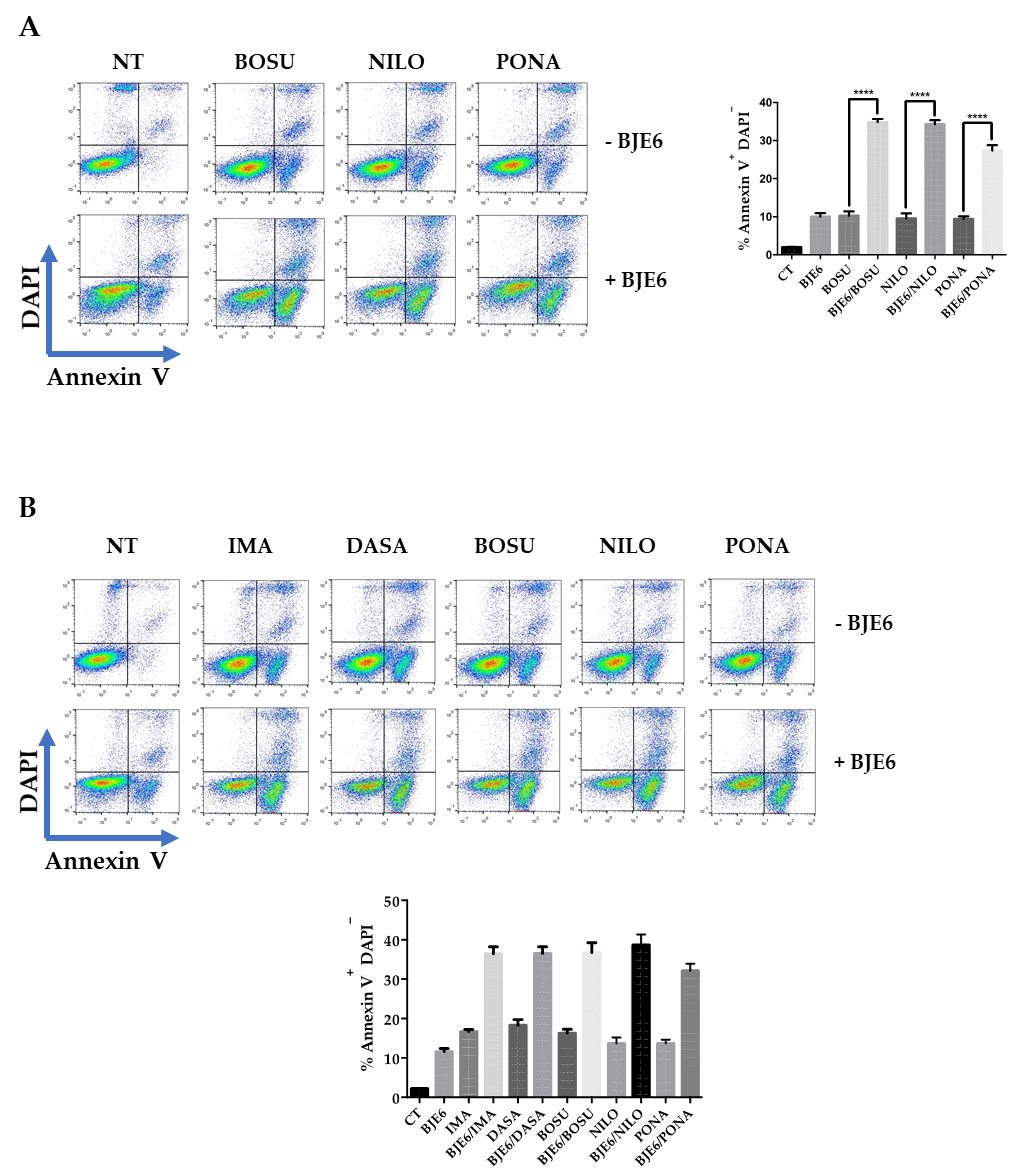


**Figure S10.** Inhibition of PKCδ enhanced early apoptosis induced by TKIs in CML cells. (**A**) K562 cells and (**B**) LAMA-84 cells were stimulated or not for 24 h with 0.5 µM BJE6-106 (BJE6) in combination or not with indicated TKIs. Cytometric analysis after Annexin V/DAPI labelling. Histograms represent early apoptosis.

Table Relative to Fig S10A

|  | **CT** | **BJE6** | **BOSU** | **BJE6/BOSU** | **NILO** | **BJE6/NILO** | **PONA** | **BJE6/PONA** |  | |
| --- | --- | --- | --- | --- | --- | --- | --- | --- | --- | --- |
| Relative Cell Number | 642.20 | 3164.76 | 3245.69 | 10994.15 | 3020.76 | 10815.47 | 2976.62 | 8618.74 |  |  |
| Standard Deviation | 24.49 | 290.40 | 357.94 | 244.92 | 415.26 | 333.70 | 209.70 | 452.93 |  |  |

Table relative to Fig S10B

|  | **CT** | **BJE6** | **IMA** | **BJE6/IMA** | **DASA** | **BJE6/DASA** |
| --- | --- | --- | --- | --- | --- | --- |
| Relative Cell Number | 706.51 | 3697.26 | 5322.79 | 11612.39 | 5843.38 | 11644.26 |
| Standard Deviation | 18.12 | 248.93 | 168.65 | 574.00 | 439.72 | 546.81 |

|  | **BOSU** | **BJE6/BOSU** | **NILO** | **BJE6/NILO** | **PONA** | **BJE6/PONA** |
| --- | --- | --- | --- | --- | --- | --- |
| Relative Cell Number | 5195.29 | 11708.01 | 4355.97 | 12334.85 | 4355.97 | 10252.48 |
| Standard Deviation | 313.91 | 810.31 | 496.16 | 848.68 | 314.45 | 566.88 |


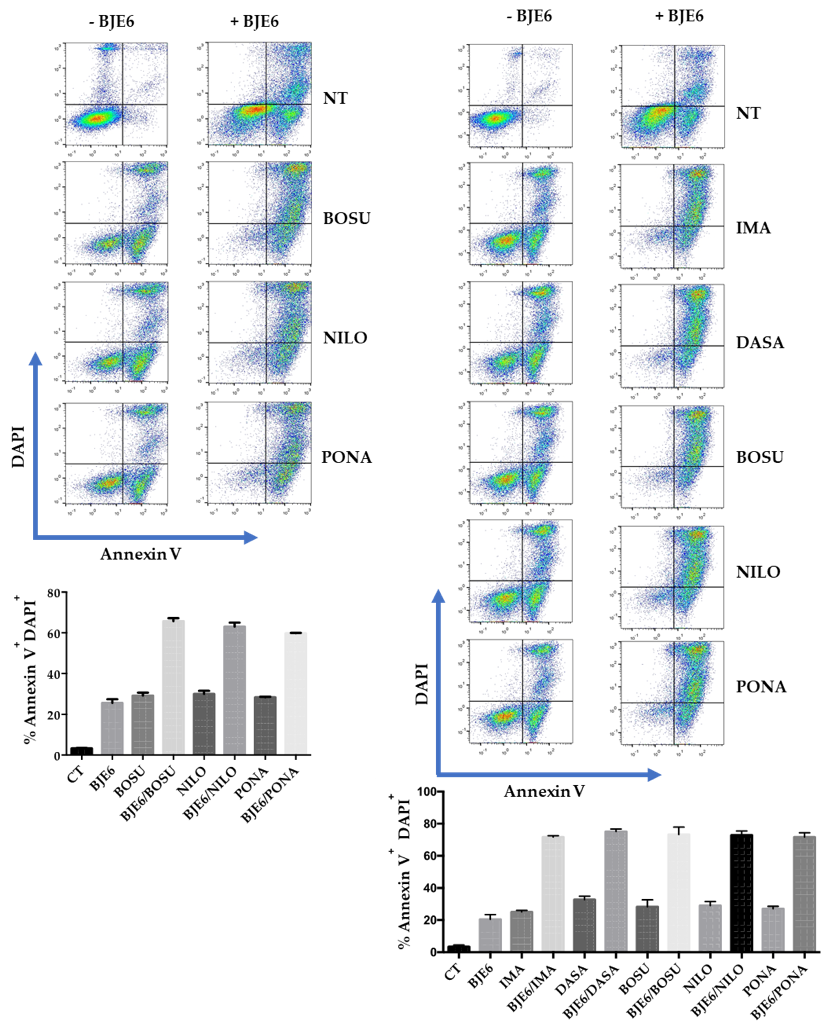


**Figure S11.** Apoptotic events induced by combinations of PKCδ and BCR-ABL inhibitors in K562 cells. K562 cells (left) and LAMA-84 cells (right) were stimulated or not with 0.5 µM BJE6 in combination or not with 1 µM IMA or 2 nM DASA, 10 nM BOSU, 2 nM NILO, or 2 nM PONA for 48 h. Cytometric analysis after Annexin V/DAPI labelling (top panel). Histogram for Annexin V + DAPI + cells (bottom panel).

**K562**

|  | **CT** | **BJE6** | **BOSU** | **BJE6/BOSU** | **NILO** | **BJE6/NILO** | **PONA** | **BJE6/PONA** |  | |
| --- | --- | --- | --- | --- | --- | --- | --- | --- | --- | --- |
| Relative Cell Number | 1092.35 | 8482.56 | 9697.51 | 21780.74 | 9951.54 | 20908.18 | 9399.29 | 19825.77 |  |  |
| Standard Deviation | 70.86 | 602.83 | 473.64 | 507.22 | 513.68 | 645.07 | 95.65 | 50.61 |  |  |

**LAMA-84**

|  | **CT** | **BJE6** | **IMA** | **BJE6/IMA** | **DASA** | **BJE6/DASA** |
| --- | --- | --- | --- | --- | --- | --- |
| Relative Cell Number | 1058.68 | 6476.75 | 7934.81 | 22737.31 | 10354.35 | 23815.01 |
| Standard Deviation | 140.18 | 378.15 | 131.96 | 101.89 | 276.93 | 203.78 |

|  | **BOSU** | **BJE6/BOSU** | **NILO** | **BJE6/NILO** | **PONA** | **BJE6/PONA** |
| --- | --- | --- | --- | --- | --- | --- |
| Relative Cell Number | 8959.68 | 23233.90 | 9202.69 | 23085.98 | 8579.32 | 22705.61 |
| Standard Deviation | 554.77 | 587.03 | 323.76 | 332.94 | 191.06 | 347.70 |


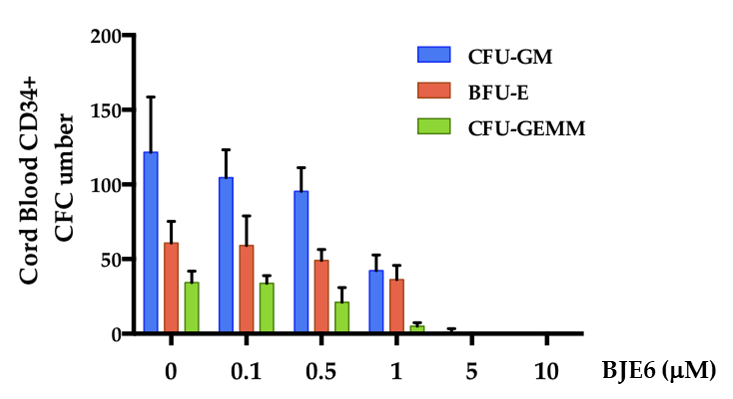


**Figure S12.** Evaluation of BJE6 toxicity in healthy CD34^+^ cells. Clonogenic capacity of cord blood CD34^+^ cells examined after 14 days with indicated concentrations of BJE6. Error bars represent SD for 3 biological replicates.


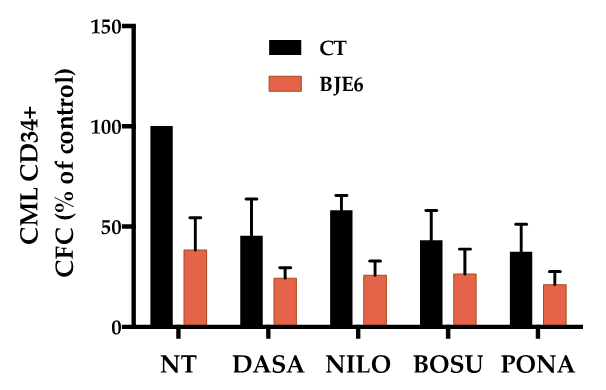


**Figure S13.** Dual inhibition of PKCδ and BCR-ABL inhibits CML CD34^+^ clonogenicity. Clonogenic capacity of primary CD34^+^ cells from diagnosed CML patients treated with DMSO (CT), 2 nM DASA, 2 nM NILO, 10 nM BOSU, or 2 nM PONA combined or not with 0.1 µM BJE6 was examined after 14 days. Results are expressed as % of colonies in each well without treatment (182 ± 34%).

**Table S1.** Relative to Fig 4B. Proportions of mitochondrial depolarized cells (ΔΨm) are expressed as a percentage of the total population of cells for each condition.

|  | **CT** | **FCCP** | **BJE6** | **IMA** | **BJE6/IMA** | **DASA** | **BJE6/DASA** |
| --- | --- | --- | --- | --- | --- | --- | --- |
| Relative Cell Number | 973.27 | 21135.43 | 3503.79 | 4938.09 | 12970.17 | 5378.62 | 12755.02 |
| Standard Deviation | 46.94 | 895.72 | 452.75 | 124.21 | 1291.96 | 81.31 | 832.11 |

**Table S2.** Relative to Fig 4C. Proportions of early apoptosis (Annexin V + DAPI –) are expressed as a percentage of the total population of cells for each condition.

|  | **CT** | **BJE6** | **IMA** | **BJE6/IMA** | **DASA** | **BJE6/DASA** |
| --- | --- | --- | --- | --- | --- | --- |
| Relative Cell Number | 847.76 | 2535.71 | 329.66 | 11187.91 | 428219 | 11122.86 |
| Standard Deviation | 39.20 | 39.34 | 75.10 | 181.08 | 49.68 | 266.212 |

**Table S3.** Relative to Fig 4C. Proportions of late apoptosis (Annexin V + DAPI +) are expressed as a percentage of the total population of cells for each condition.

|  | **CT** | **BJE6** | **IMA** | **BJE6/IMA** | **DASA** | **BJE6/DASA** |
| --- | --- | --- | --- | --- | --- | --- |
| Relative Cell Number | 1242.80 | 2130.36 | 2734.59 | 4657.83 | 3616.79 | 4614.90 |
| Standard Deviation | 11.60 | 133.89 | 90.74 | 177.32 | 209.48 | 237.32 |
