# Supplementary material for: Combination of PKCδ Inhibition with Conventional TKI Treatment to Target CML Models
Source: Cancers (Basel). 2021 Apr 2;13(7):1693. doi: 10.3390/cancers13071693 (PMC8038300; doi:10.3390/cancers13071693)
Supplement: Supplementary file 1 [file cancers-13-01693-s001.zip › cancers-1161399-supplementary-proof/Figure S14.pdf]

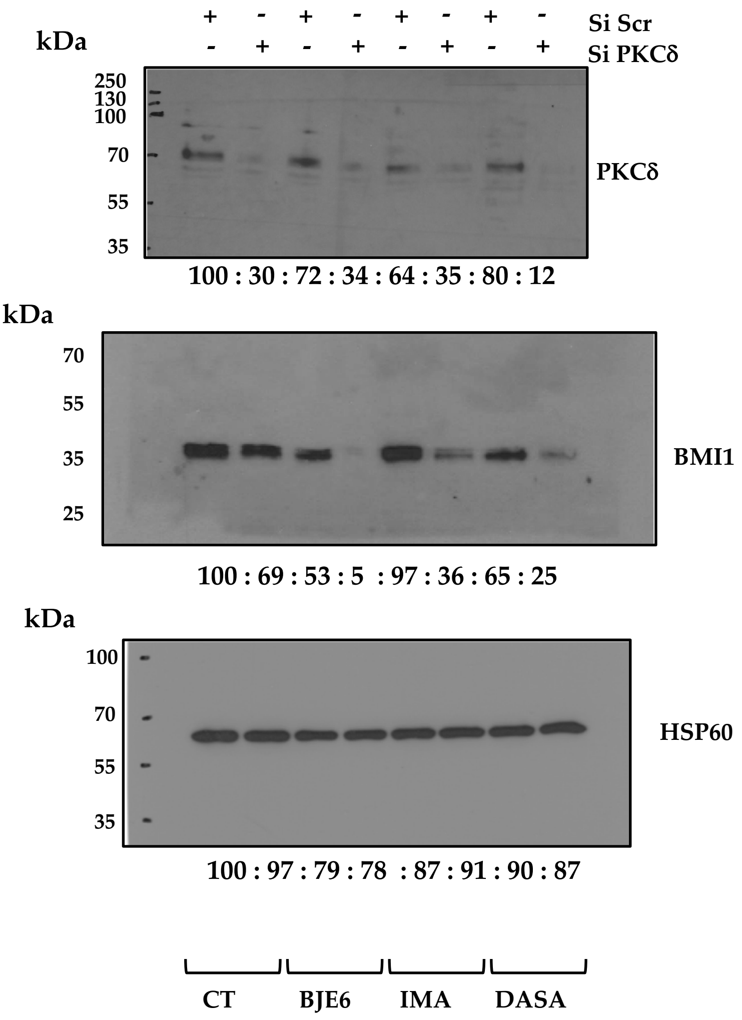
Si Scr
Si PKCδ
kDa
250
130
100
70
55
35
PKCδ
100 : 30 : 72 : 34 : 64 : 35 : 80 : 12
kDa
70
55
35
25
BMI1
100 : 69 : 53 : 5 : 97 : 36 : 65 : 25
kDa
100
70
55
35
HSP60
100 : 97 : 79 : 78 : 87 : 91 : 90 : 87
CT
BJE6
IMA
DASA

Figure 1D

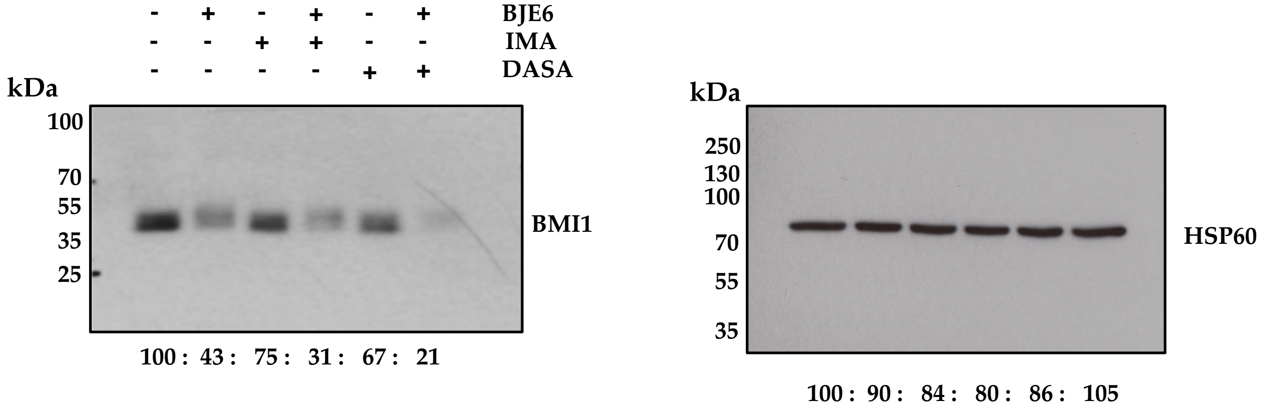

Figure 2A

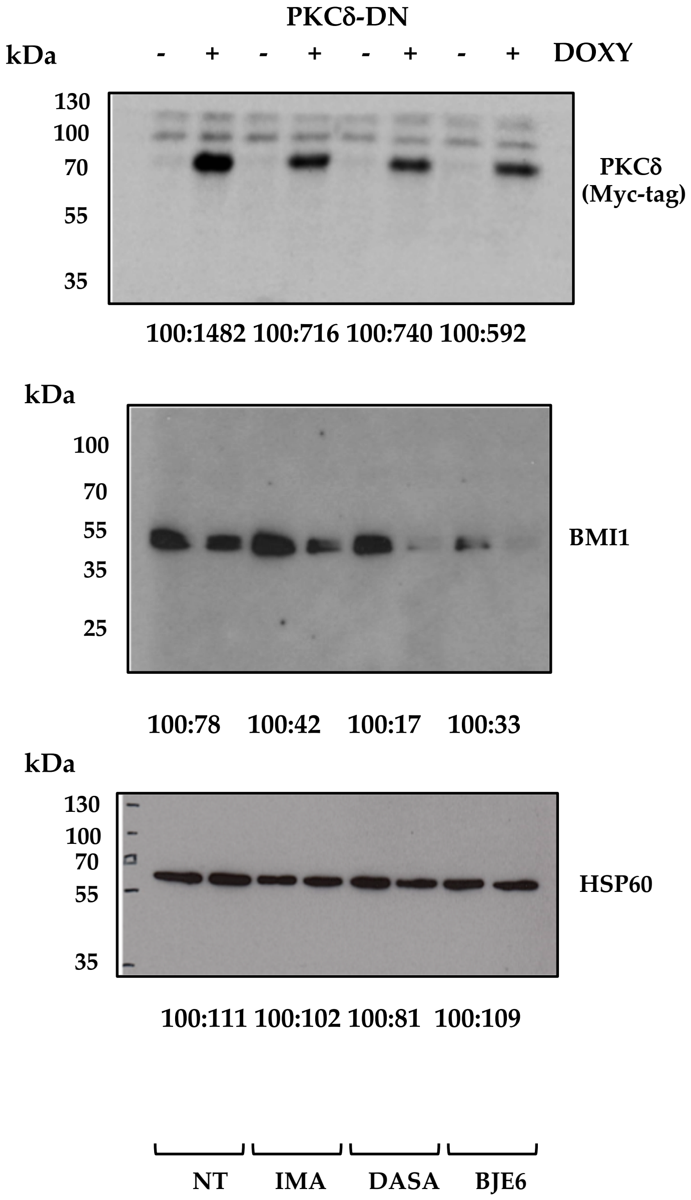

Figure 2B

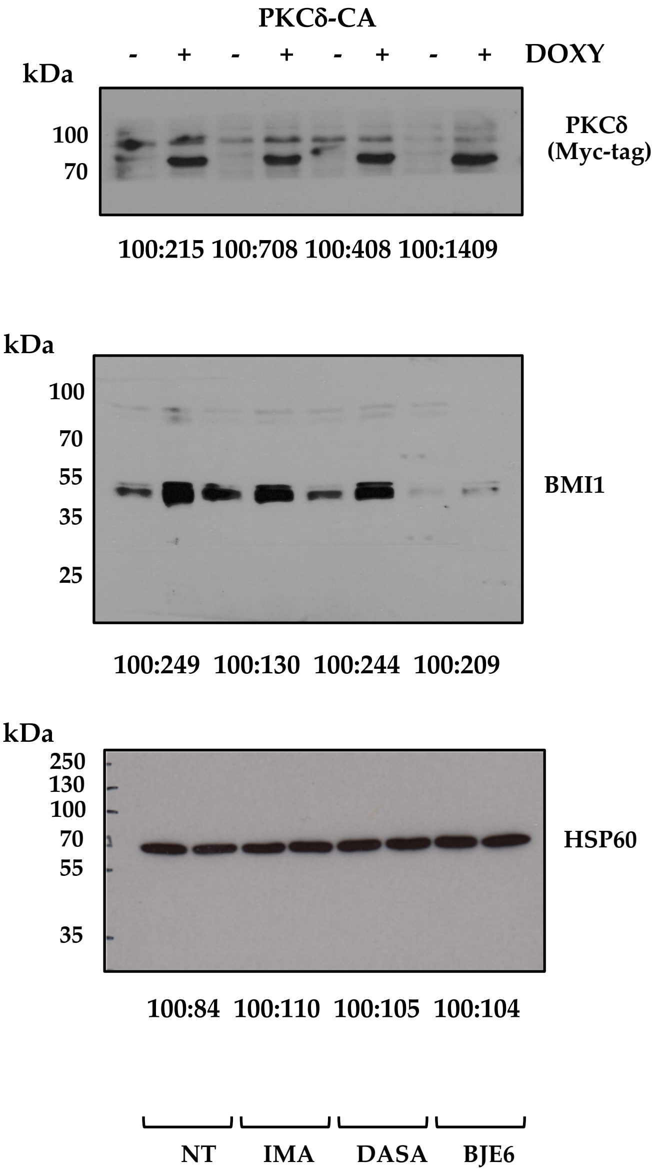

Figure 2D

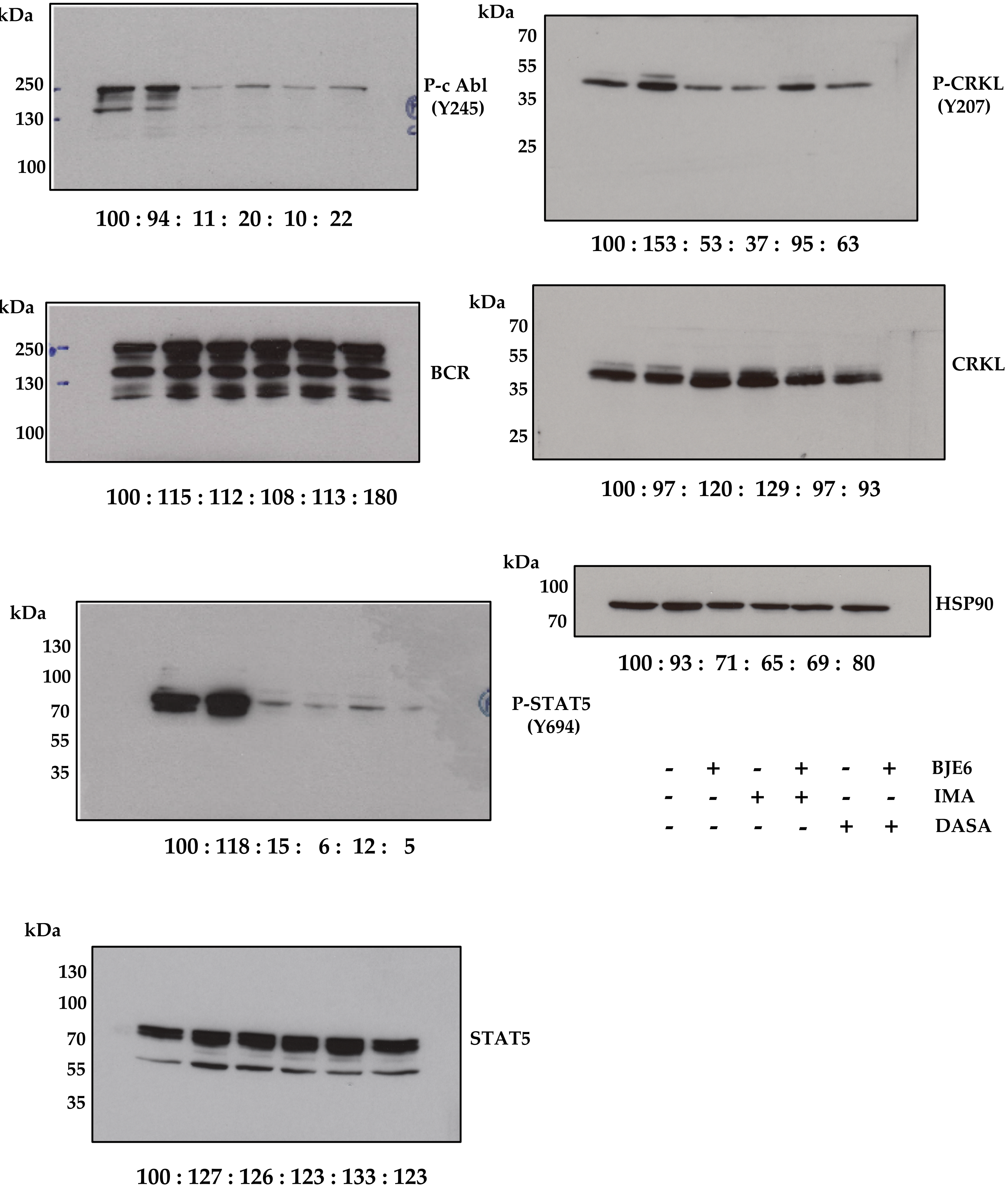
kDa
250
130
100
P-c Abl (Y245)
100 : 94 : 11 : 20 : 10 : 22
kDa
70
55
35
25
P-CRKL (Y207)
100 : 153 : 53 : 37 : 95 : 63
kDa
250
130
100
BCR
100 : 115 : 112 : 108 : 113 : 180
kDa
70
55
35
25
CRKL
100 : 97 : 120 : 129 : 97 : 93
kDa
100
70
HSP90
100 : 93 : 71 : 65 : 69 : 80
kDa
130
100
70
55
35
P-STAT5 (Y694)
100 : 118 : 15 : 6 : 12 : 5
- + - + - + BJE6
- - + + - - IMA
- - - - + + DASA
kDa
130
100
70
55
35
STAT5
100 : 127 : 126 : 123 : 133 : 123

Figure 4A

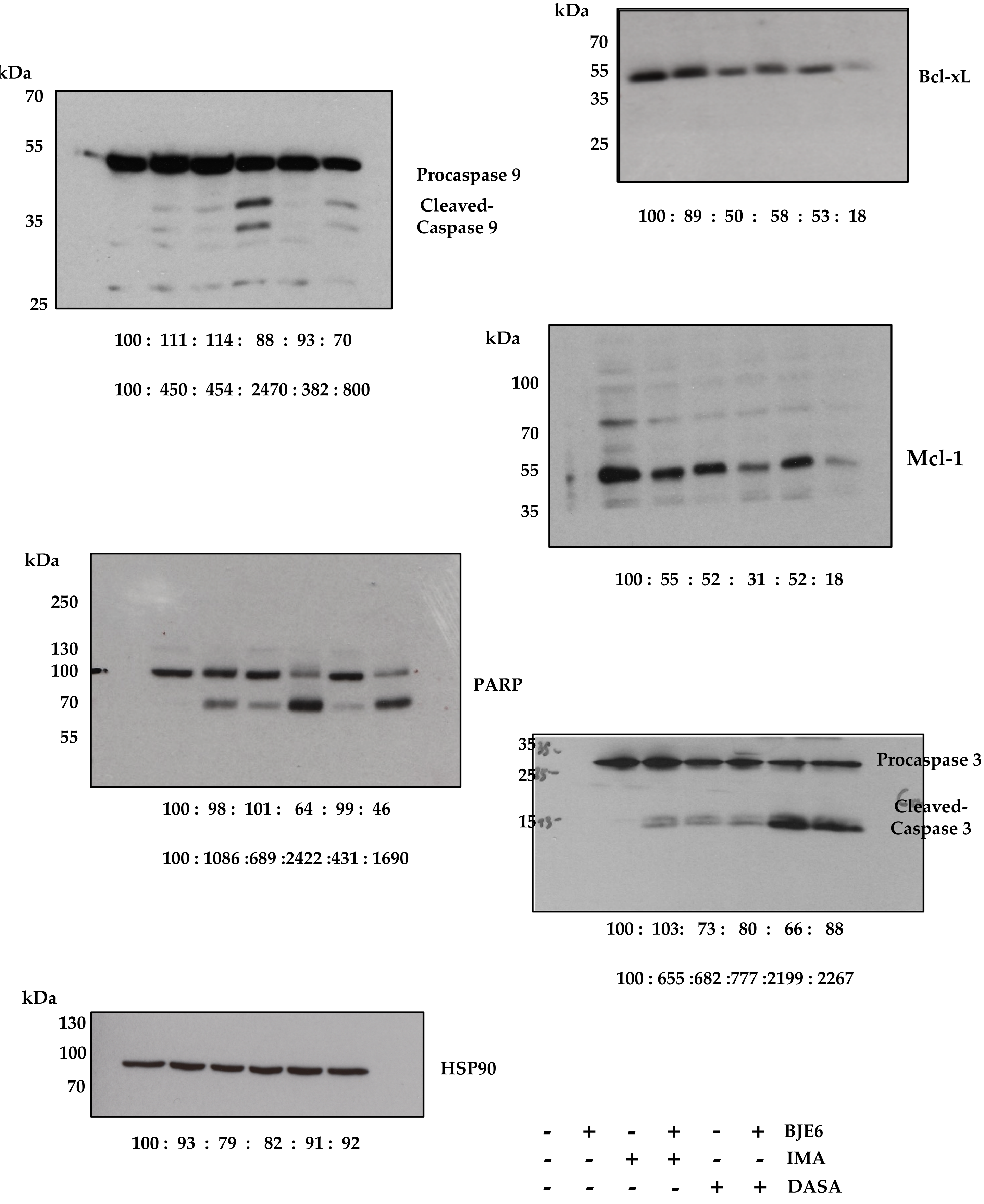

kDa
70
55
35
25
Procaspase 9
Cleaved-
Caspase 9
100 : 111 : 114 : 88 : 93 : 70
100 : 450 : 454 : 2470 : 382 : 800
kDa
70
55
35
25
Bcl-xL
100 : 89 : 50 : 58 : 53 : 18
kDa
100
70
55
35
Mcl-1
100 : 55 : 52 : 31 : 52 : 18
kDa
250
130
100
70
55
PARP
100 : 98 : 101 : 64 : 99 : 46
100 : 1086 :689 :2422 :431 : 1690
35
25
15
Procaspase 3
Cleaved-
Caspase 3
100 : 103: 73 : 80 : 66 : 88
100 : 655 :682 :777 :2199 : 2267
kDa
130
100
70
HSP90
100 : 93 : 79 : 82 : 91 : 92
- + - + - + BJE6
- - + + - - IMA
- - - - + + DASA

Figure 4D

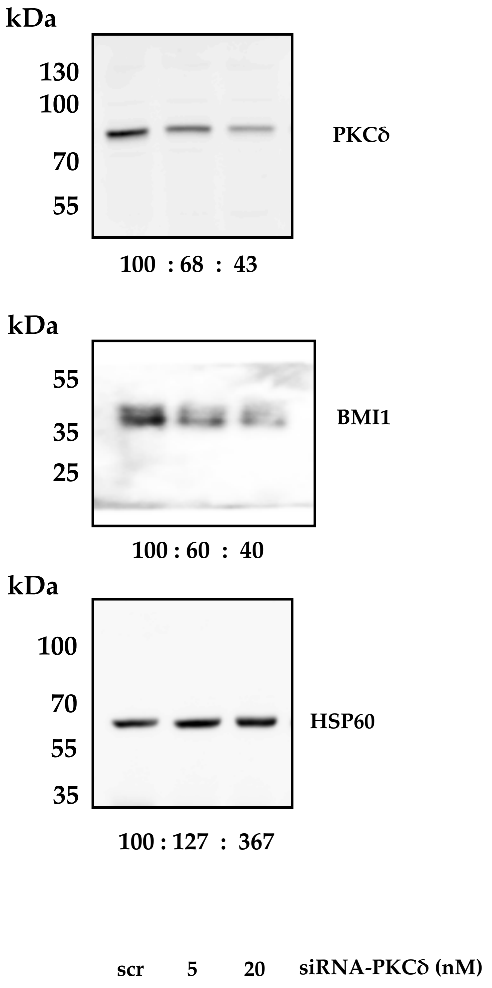
kDa
130
100
70
55
PKCδ
100 : 68 : 43
kDa
55
35
25
BMI1
100 : 60 : 40
kDa
100
70
55
35
HSP60
100 : 127 : 367
scr
5
20
siRNA-PKCδ (nM)

Figure S2

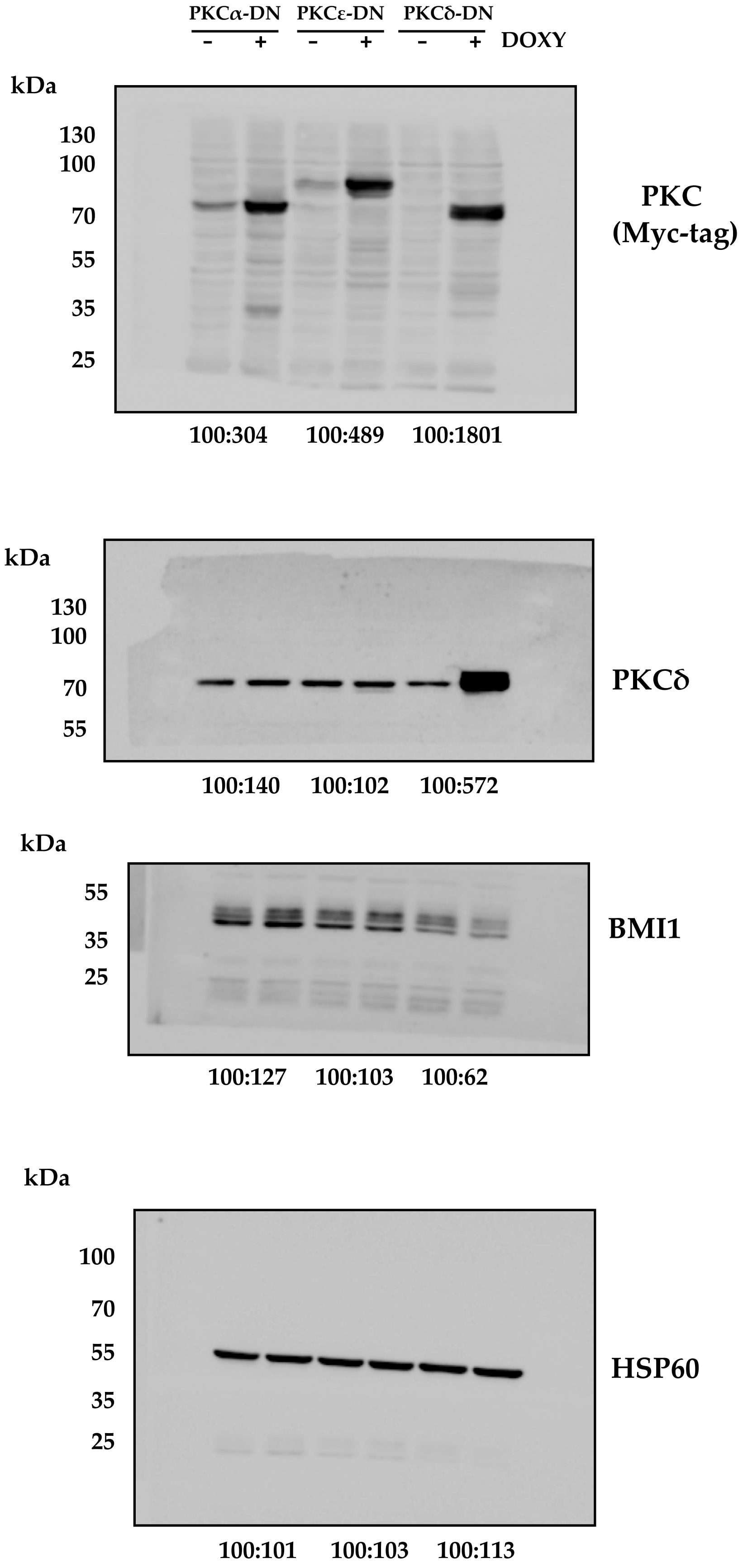
PKCα-DN
PKCε-DN
PKCδ-DN
- + - + - + DOXY
kDa
130
100
70
55
35
25
PKC (Myc-tag)
100:304
100:489
100:1801
kDa
130
100
70
55
PKCδ
100:140
100:102
100:572
kDa
55
35
25
BMI1
100:127
100:103
100:62
kDa
100
70
55
35
25
HSP60
100:101
100:103
100:113

Figure S3

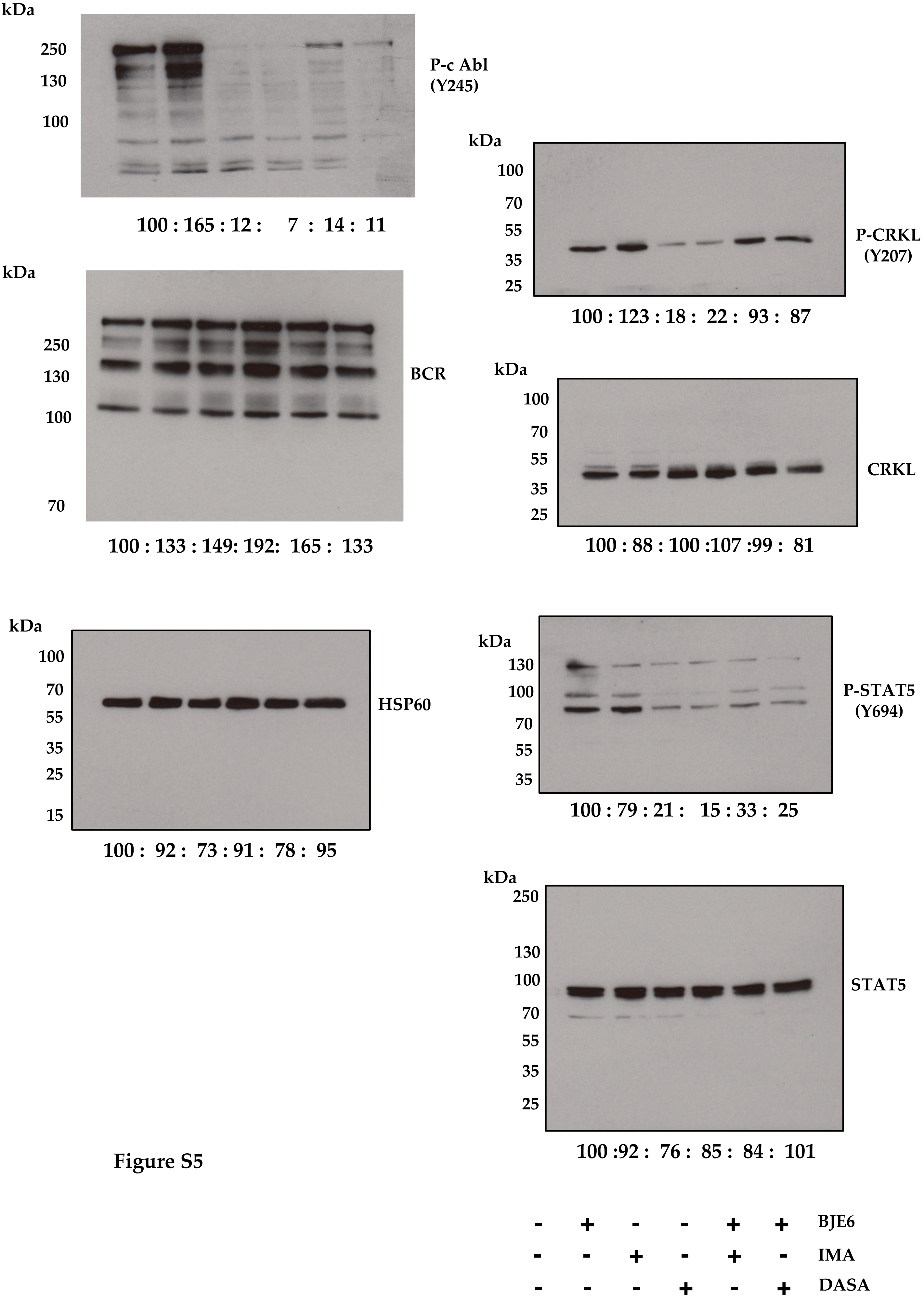
kDa
250
130
100
P-c Abl
(Y245)
100 : 165 : 12 : 7 : 14 : 11
kDa
100
70
55
35
25
P-CRKL
(Y207)
100 : 123 : 18 : 22 : 93 : 87
kDa
250
130
100
70
BCR
100 : 133 : 149: 192: 165 : 133
kDa
100
70
55
35
25
CRKL
100 : 88 : 100 :107 :99 : 81
kDa
100
70
55
35
25
15
HSP60
100 : 92 : 73 : 91 : 78 : 95
kDa
130
100
70
55
35
P-STAT5
(Y694)
100 : 79 : 21 : 15 : 33 : 25
kDa
250
130
100
70
55
35
25
STAT5
100 :92 : 76 : 85 : 84 : 101
- + - - + + BJE6
- - + - + - IMA
- - - + - + DASA

Figure S5

Figure S8

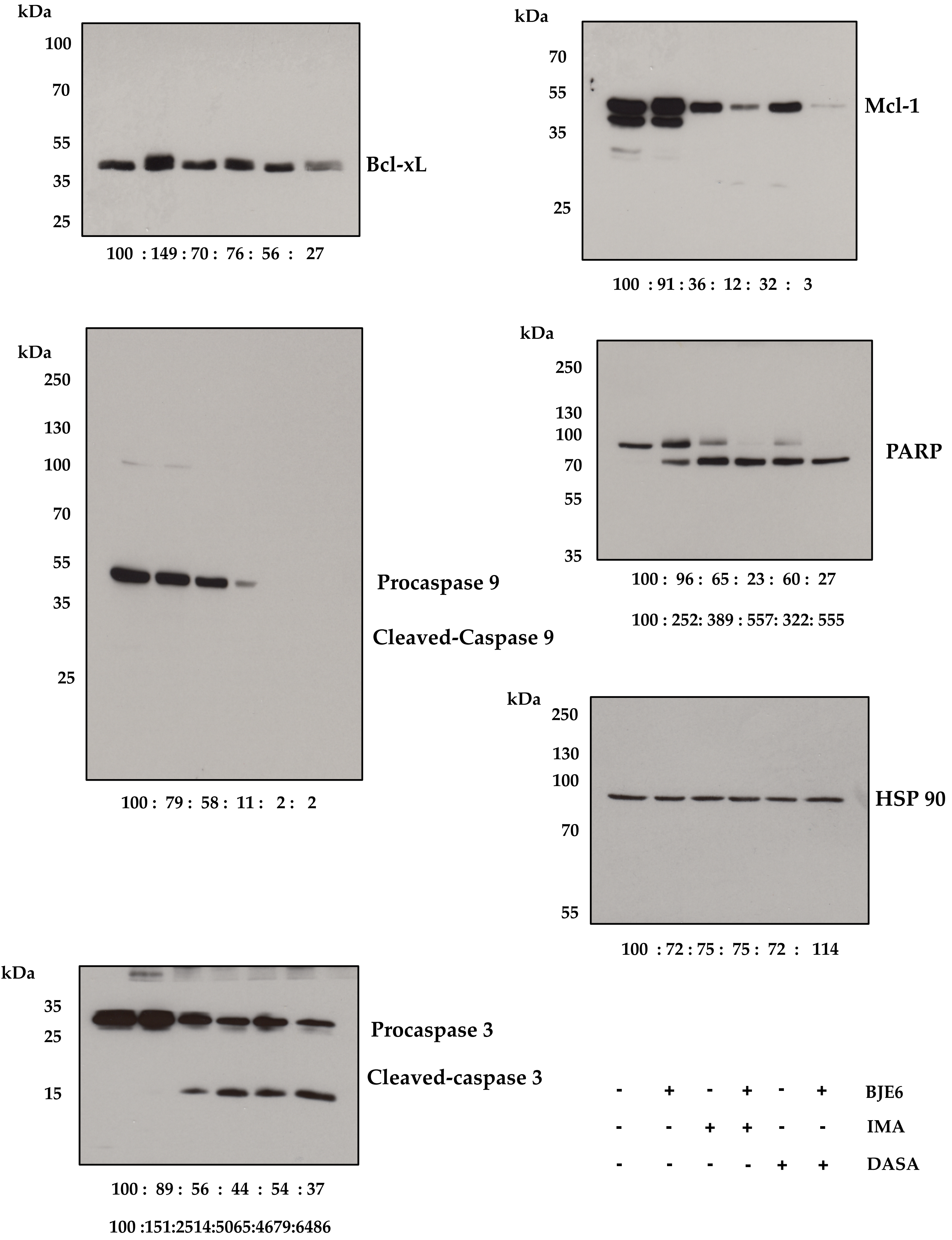

kDa
100
70
55
35
25
Bcl-xL
100 : 149 : 70 : 76 : 56 : 27
kDa
70
55
35
25
Mcl-1
100 : 91 : 36 : 12 : 32 : 3
kDa
250
130
100
70
55
35
25
Procaspase 9
Cleaved-Caspase 9
100 : 79 : 58 : 11 : 2 : 2
kDa
250
130
100
70
55
35
PARP
100 : 96 : 65 : 23 : 60 : 27
100 : 252: 389 : 557: 322: 555
kDa
250
130
100
70
55
HSP 90
100 : 72 : 75 : 75 : 72 : 114
kDa
35
25
15
Procaspase 3
Cleaved-caspase 3
100 : 89 : 56 : 44 : 54 : 37
100 :151:2514:5065:4679:6486
- + - + - + BJE6
- - + + - - IMA
- - - - + + DASA

Figure S8

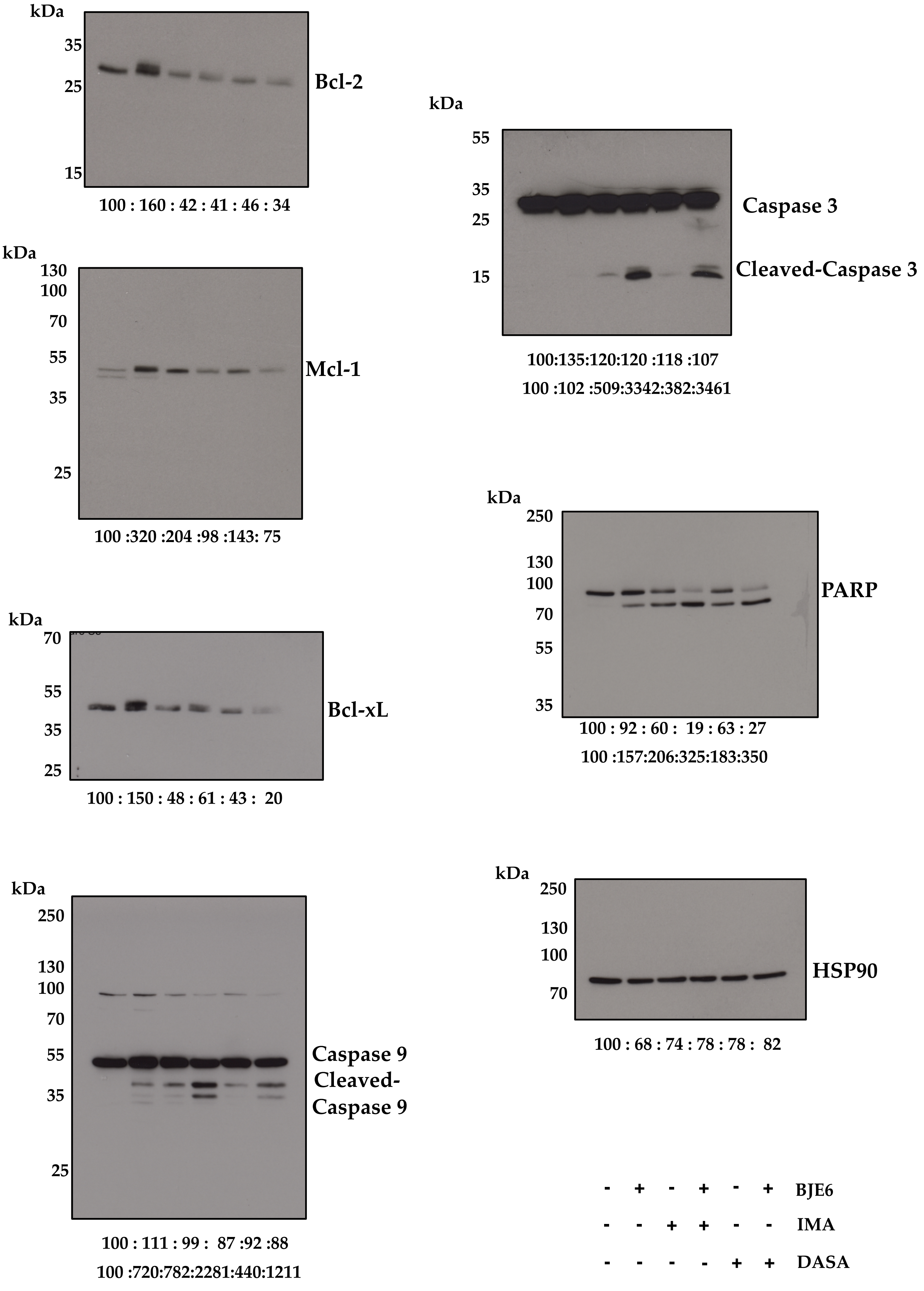

kDa
35
25
15
Bcl-2
100 : 160 : 42 : 41 : 46 : 34
kDa
130
100
70
55
35
25
Mcl-1
100 :320 :204 :98 :143: 75
kDa
70
55
35
25
Bcl-xL
100 : 150 : 48 : 61 : 43 : 20
kDa
250
130
100
70
55
35
25
Caspase 9
Cleaved-
Caspase 9
100 : 111 : 99 : 87 :92 :88
100 :720:782:2281:440:1211
kDa
55
35
25
15
Caspase 3
Cleaved-Caspase 3
100:135:120:120 :118 :107
100 :102 :509:3342:382:3461
kDa
250
130
100
70
55
35
PARP
100 : 92 : 60 : 19 : 63 : 27
100 :157:206:325:183:350
kDa
250
130
100
70
HSP90
100 : 68 : 74 : 78 : 78 : 82
- + - + - + BJE6
- - + + - - IMA
- - - - + + DASA
